# A role for *SETD2* loss in tumorigenesis through DNA methylation dysregulation

**DOI:** 10.1186/s12885-023-11162-0

**Published:** 2023-08-01

**Authors:** Hira Javaid, Alessandro Barberis, Olga Chervova, Isar Nassiri, Vitaly Voloshin, Yusuke Sato, Seishi Ogawa, Benjamin Fairfax, Francesca Buffa, Timothy C. Humphrey

**Affiliations:** 1 Department of Oncology, University of Oxford, Oxford, OX3 7DQ UK; 2 Nuffield Department of Surgical Sciences, University of Oxford, John Radcliffe Hospital, Oxford, OX3 9DU UK; 3 UCL Cancer Institute, University College London, London, WC1E 6DD UK; 4 Oxford Genomics Centre, Wellcome Centre for Human Genetics, Nuffield Department of Medicine, University of Oxford, Oxford, OX3 7BN UK; 5 Royal Botanic Gardens Kew, Kew Green, Richmond, TW9 3AE Surrey UK; 6 School of Biological and Behavioural Sciences, Queen Mary University of London, London, E1 4NS UK; 7 Department of Pathology and Tumor Biology, Graduate School of Medicine, Kyoto University, Kyoto, Japan; 8 The MRC Weatherall Institute of Molecular Medicine, University of Oxford, John Radcliffe Hospital/Headley Way, OX3 9DS Oxford, UK; 9 Genome Damage and Stability Centre, School of Life Sciences, University of Sussex, BN1 9RQ Brighton, UK

**Keywords:** DNA methylation, *SETD2*, H3K36me3, Renal cancer biomarker, Machine learning biomarker

## Abstract

SETD2-dependent H3 Lysine-36 trimethylation (H3K36me3) has been recently linked to the deposition of *de-novo* DNA methylation. *SETD2* is frequently mutated in cancer, however, the functional impact of *SETD2* loss and depletion on DNA methylation across cancer types and tumorigenesis is currently unknown. Here, we perform a pan-cancer analysis and show that both *SETD2* mutation and reduced expression are associated with DNA methylation dysregulation across 21 out of the 24 cancer types tested. In renal cancer, these DNA methylation changes are associated with altered gene expression of oncogenes, tumour suppressors, and genes involved in neoplasm invasiveness, including *TP53, FOXO1*, and *CDK4*. This suggests a new role for *SETD2* loss in tumorigenesis and cancer aggressiveness through DNA methylation dysregulation. Moreover, using a robust machine learning methodology, we develop and validate a 3-CpG methylation signature which is sufficient to predict *SETD2* mutation status with high accuracy and correlates with patient prognosis.

**Supplementary Information:**

The online version contains supplementary material available at 10.1186/s12885-023-11162-0.

## Introduction

DNA methylation is the addition of methyl groups to specific bases within DNA, most commonly CpG sites—cytosines followed by guanines [1]. DNA methylation is often associated with changes in gene expression; methylation in gene promoter regions usually co-occurs with silencing and methylation at gene bodies with actively transcribed genes [2, 3]. Dysregulated DNA methylation is an occurrence in every cancer type, is often an early event in the tumorigenic process, and can also be identified in pre-cancerous lesions [4–7]. As cancer cells release DNA into the blood in the form of circulating tumour DNA, such modifications have garnered considerable interest in the early cancer detection field due to their potential to be used as a non-invasive biomarker for cancer risk and early cancer detection [8–11]. However, despite there being thousands of biomarker studies published in the literature, only a few have been translated into the clinics, partially due to limitations in the methods used for biomarker development and subsequent validation and testing, resulting in models that are not generalizable and so perform poorly on new subsequent datasets [12–15].

DNA methylation can crosstalk with other epigenetic marks to establish or maintain chromatin structures [16]. For example, SETD2-dependent H3K36 trimethylation (H3K36me3) recruits the *de-novo* DNA methyltransferase DNMT3B through its PWWP domain which subsequently methylates DNA at nearby regions [17, 18]. This first occurs in active genes during development as the transcription machinery first passes along a gene [19]. While this link is well-recognised, whether loss of SETD2 and H3K36me3 can lead to subsequent loss of DNA methylation after *de-novo* methylation is established is subject to controversy. Neri et al. showed that knocking out *SETD2* led to reduced recruitment of DNMT3B and reduced gene body methylation [20]. However, in another study, depletion of H3K36me3 through *SETD2* knockdown did not lead to reduced DNA methylation [21].

Understanding the impact of loss or depletion of *SETD2* and H3K36me3 on DNA methylation is of particular clinical relevance as *SETD2* is frequently mutated in multiple cancer types, for example, in up to 16% of clear cell renal cell carcinomas (ccRCC) and can be as high as 30% in metastatic ccRCC [22, 23]. We and others have also found that H3K36me3 is frequently lost or depleted in up to 60% of metastatic ccRCC [22, 24, 25]. *SETD2* is also frequently mutated or under-expressed in several other cancer types including 15.9% of Phyllodes breast tumours, 15% of paediatric high grade gliomas, and 8–10% of pancreatic ductal adenocarcinomas [26–28]. Depletion of and H3K36me3 is associated with more aggressive tumours and worse prognosis [29–34]. Thus, understanding the impact of *SETD2* and H3K36me3 loss on DNA methylation and tumorigenesis is of biological and clinical importance.

Previously, association of *SETD2* with DNA methylation has been shown in 4 cancer types [22, 25, 26, 33–37]. These studies show a widespread hypermethylation change in cancer, however since *SETD2* alterations is linked with reduced H3K36me3, we predicted it would also lead to gene body hypomethylation through reduced H3K36me3-dependent DNMT3B recruitment. We, thus, expanded the study to include more cancer types including those that show infrequent *SETD2* mutations to explore if *SETD2* downregulation also impacted on DNA methylation. Further, previous studies have not explored the effect of these SETD2-dependent methylation changes on gene expression or tumorigenic process which remains a research gap. Loss of *SETD2* and DNA methylation changes can both be early events in cancers, thus we wished to explore the role of *SETD2* loss on DNA methylation dysregulation and tumorigenesis, which has not been studied before.

In this study, we performed a pan-cancer analysis to look at the effect of *SETD2* mutation and depletion in 24 cancer types using The Cancer Genome Atlas (TCGA) data and found that both *SETD2* alternative variants and reduced expression are associated with characteristic DNA methylation changes in 21 out of the 24 cancer types tested. Importantly, we find that in renal cancer, these DNA methylation changes are significantly correlated with gene expression changes in oncogenes and tumour suppressors, including *TP53, FOXO1*, and *CDK4*. Genes with dysregulated expression that correlated with SETD2-dependent DNA methylation changes are enriched for tumorigenic processes such as neoplasm invasiveness, suggesting a new role for SETD2 in tumorigenesis and cancer aggressiveness through DNA methylation dysregulation. Further, we develop a renal cancer signature of *SETD2* loss using a unique machine-learning approach comprising multiple random sampling and repeated cross-validation, and successfully validated our biomarker in an independent Japanese renal cancer dataset. We also show that our CpG signature is associated with prognosis. Our approach allows for a more accurate estimate of biomarker performance in external datasets and thus, we anticipate this approach will be useful in developing more readily clinically-translatable biomarkers in future studies.

## Results

### ***SETD2*** alternative variants and depletion is associated with DNA methylation changes in multiple cancer types

SETD2 is a histone H3K36me3 methyltransferase and loss of SETD2 function is associated with a loss or depletion of H3K36me3 in somatic cells [25]. However, the impact of SETD2 loss of function on methylation has only been studied in a few cancers, namely renal, glioma, and GI stromal tumours [22, 26, 35, 37]. However, a pan-cancer analysis of the impact and functional characterisation of SETD2 loss and depletion on DNA methylation is lacking. To further explore the relationship between *SETD2* status and DNA methylation profiles we used TCGA data to assess the impact of *SETD2* mutation and copy number variation in 12 cancer types where *SETD2* alterations were present in greater than 3% of the samples and methylation data was available. All types of alternative variants, including single nucleotide variants and copy number variants, were included as cases. However, samples with *SETD2* amplification were excluded as they may result in higher levels of *SETD2* and thus H3K36me3. Samples with fusions were included as the majority of fusions cause a loss of function in *SETD2*. *SETD2* WT samples are referred to as control group.

Table 1 shows that all 13 cancer types tested show differentially methylated CpG sites when comparing *SETD2* cases to *SETD2* controls. There was no significant age difference in the *SETD2* cases and *SETD2* control groups across all cancers, except lung adenocarcinoma.

**Table 1:**
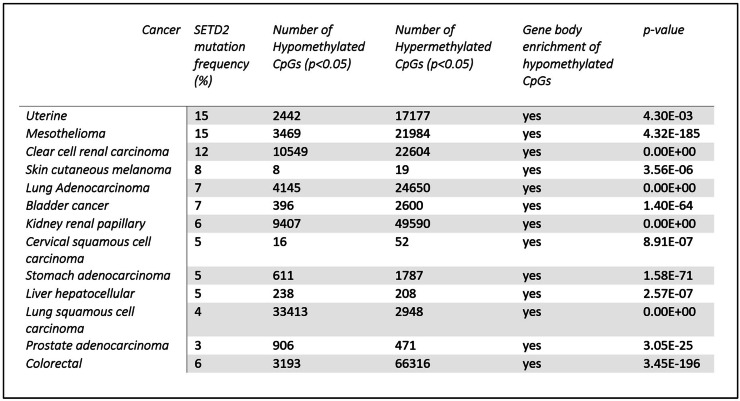
Differentially methylated CpGs for each cancer type with over 3% *SETD2* mutations

**Fig. 1:**
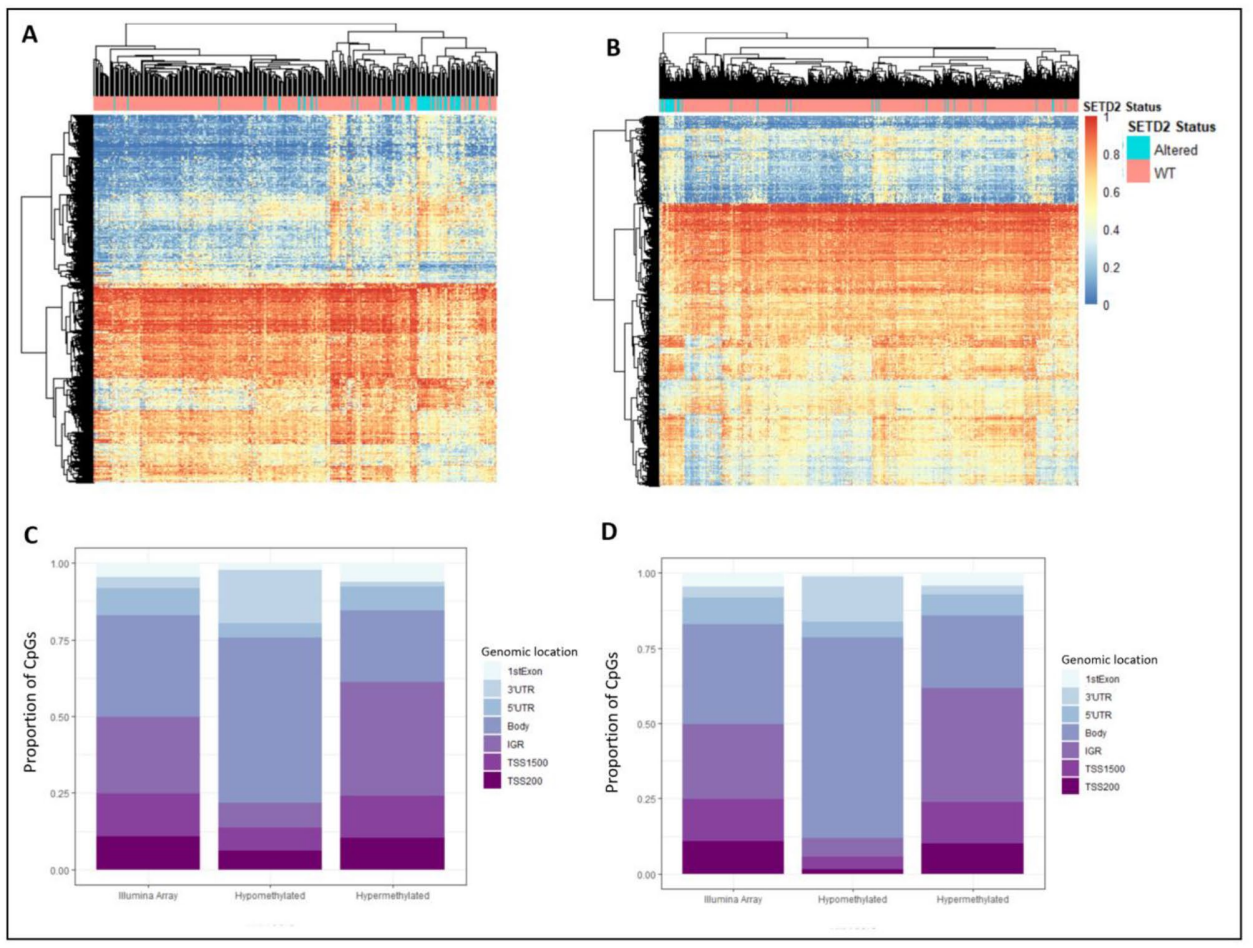
Performing a differential methylation analysis comparing *SETD2* cases and *SETD2* control cancer samples in (**A**) clear cell renal cell carcinoma and (**B**) lung adenocarcinoma shows a large number of differentially methylated CpGs. The top 10% of differentially methylated CpG sites, i.e. CpGs with the greatest ∆β values are shown in the heatmap. Unsupervised hierarchical clustering was carried out using complete-linkage clustering and distance measure is given by the Euclidean distance. Hypomethylated CpGs in both (**C**) papillary renal cell carcinoma and (**D**) lung adenocarcinoma show strong enrichment in gene bodies (p < 0.05) whereas in comparison, hypermethylated CpGs in papillary renal cell carcinoma and lung adenocarcinoma are more enriched in promoter regions and transcription start sites; Illumina array shows the distribution of CpGs in the entire 450k methylation array; β-value represents level of methylation; 3’UTR: 3’ untranslated region, 5’UTR: 5’ untranslated region; TSS1500: 1500 bp upstream of transcription start site, TSS200: 200 bp upstream of transcription start site.

We examined the top 10% of differentially methylated CpG sites in clear cell renal cell carcinoma and lung adenocarcinoma based on the most variable β-value change (∆β) (Fig. 1A and B). We find that the majority of differentially methylated CpG sites were hypermethylated; however, there is a considerable number of hypomethylated CpGs in each cancer type. The majority of hypomethylated CpGs in *SETD2* cases compared to *SETD2* controls were present in the gene bodies (Fig. 1C and D) whereas hypermethylated CpGs do not show a gene body enrichment. This gene body enrichment of hypomethylated CpGs was seen in all cancers. (Table 1 and supplementary Fig. 1).

To study the effect of loss or depletion of H3K36me3 in cancer types that had a *SETD2* mutation frequency of less than 3%, we used *SETD2* expression data to group samples into high and low *SETD2* groups. Samples with the lowest and highest quartile of *SETD2* expression based on mRNA-Seq were compared. 9 out of the 12 cancer types studied showed differential methylation between low and high *SETD2* expressing samples. Table 2 shows the DNA methylation changes found in each of these cancer types. Of these, breast cancer, , head and neck cancers, and sarcoma demonstrate the greatest DNA methylation changes. Adrenocortical carcinoma, cholangiocarcinoma, and thymomas show no differentially methylated DNA sites although the smaller sample size in these cancers may be a contributing factor.

**Table 2:**
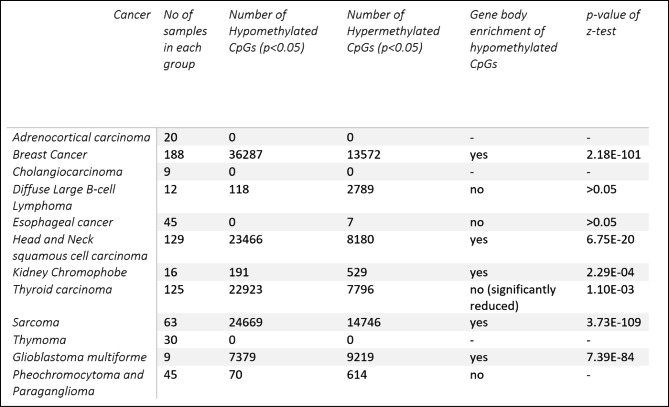
Differentially methylated CpGs when comparing samples with lowest and highest quartile of *SETD2* expression

**Fig. 2:**
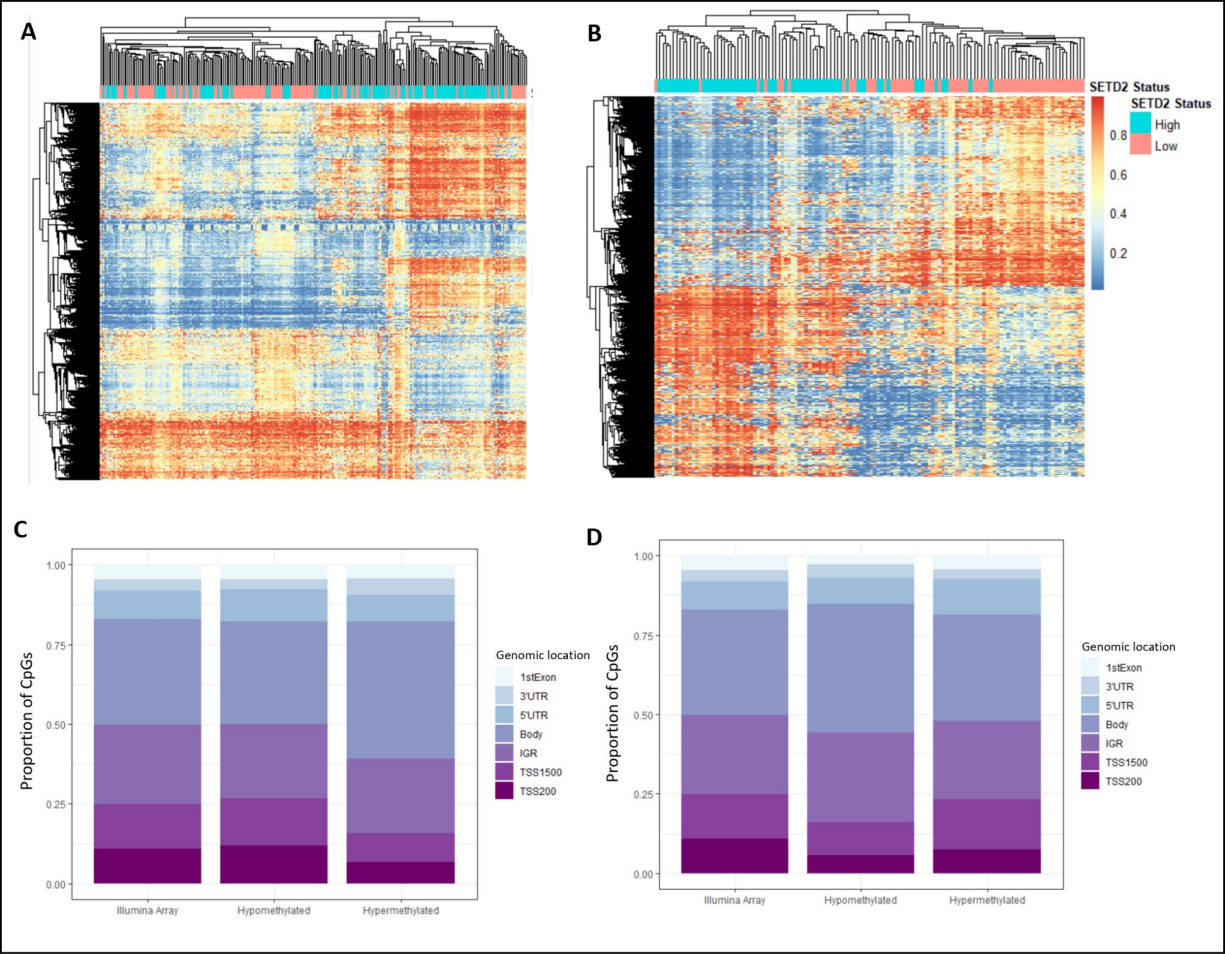
Top 10% of the differentially methylated CpGs in several cancers such as (**A**) thyroid cancer and (**B**) sarcoma when comparing tumour samples with the highest and lowest quartile of *SETD2* expression, clustered using the McQuitty clustering method and distance measure given by the Euclidean distance. The genomic distribution of hypomethylated CpGs in gene bodies in (**C**) thyroid cancer is significantly lower than expected whereas (**D**) in sarcoma, hypomethylated CpGs show a strong gene body enrichment; Illumina array shows the distribution of CpGs in the entire 450k methylation array; β-value represents level of methylation; 3’UTR: 3’ untranslated region, 5’UTR: 5’ untranslated region; TSS1500: 1500 bp upstream of transcription start site, TSS200: 200 bp upstream of transcription start site.

Figure 2 A and 2B displays the top 10% of differentially methylated CpG sites, based on ∆β, in thyroid cancer and sarcoma respectively. 4 of the 9 differentially methylated cancers tested showed greater hypomethylation than hypermethylation, and 5 showed greater hypermethylation (Table 2). 5 out of the 9 cancer types with differential methylation showed an enrichment of hypomethylated CpGs in the gene body regions (Fig. 2D, p < 0.05, Table 2). Of the remaining 4, thyroid carcinoma showed a significant reduction in the proportion of hypomethylated CpGs in the gene body (Fig. 2C, p < 0.05) whereas diffuse large B-cell lymphoma and esophageal cancer, and pheochromocytoma and paraganglioma showed no significant difference. Thus, our above analyses show that both *SETD2* alternative variants and downregulation lead to gene body hypomethylation in almost all cancer types tested. This suggests that these SETD2-dependent DNA methylation changes are due to loss of H3K36me3 which occurs in the gene body.

### VHL mutations confound the effect of ***SETD2*** loss on DNA methylation alterations in renal cancer

Loss of *SETD2* has previously been shown to affect DNA methylation in renal cancer. However, we noted that a vast majority of these samples have *VHL, BAP1,* and *PBRM1* mutations or copy number variations which are also known to impact on DNA methylation [36, 38]. Supplementary Table 2 shows that these mutations are significantly correlated with the presence of *SETD2* mutations in this tumour type.

**Fig. 3:**
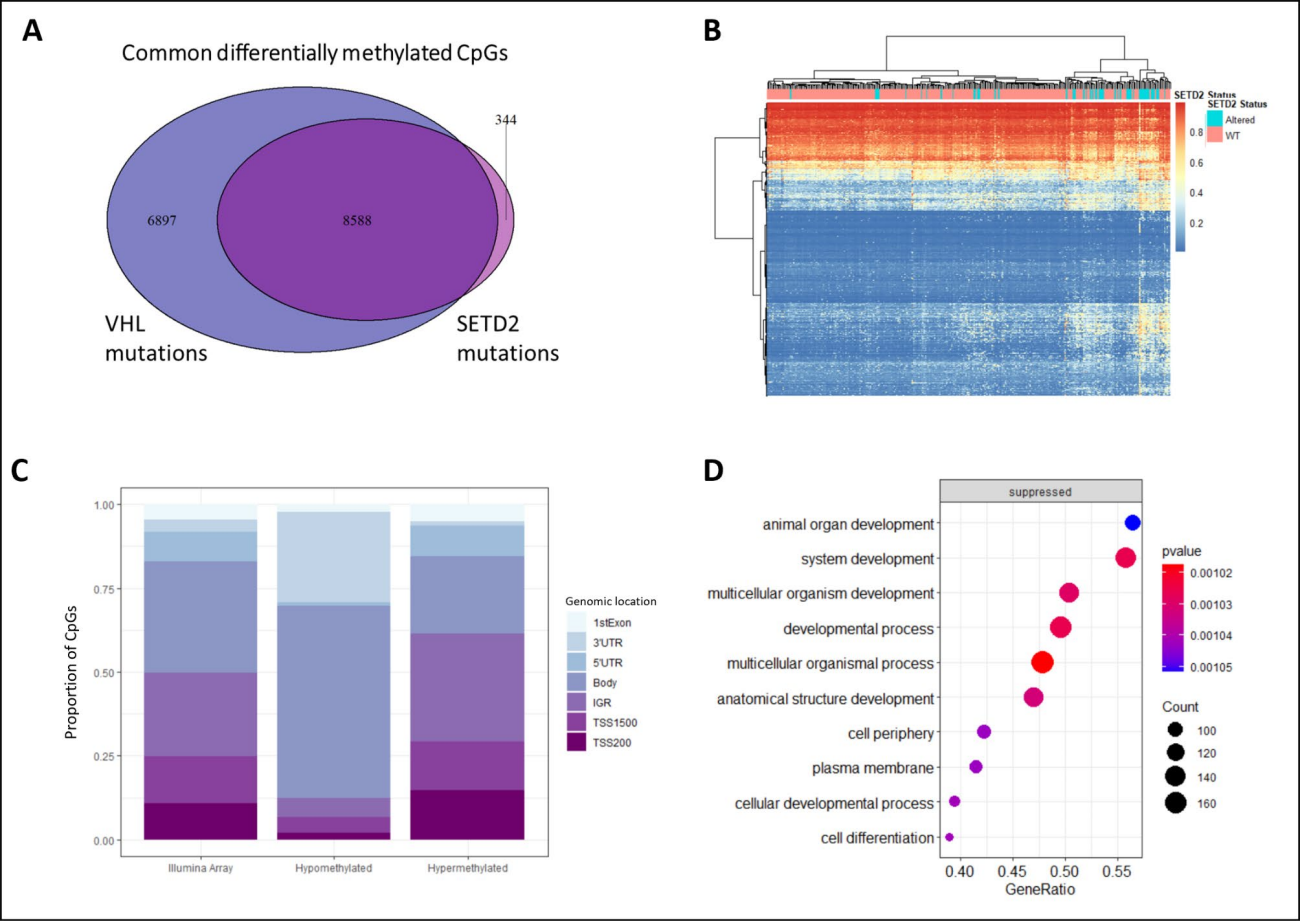
(**A**) A large overlap is seen in the differentially methylated CpGs in *SETD2* cases and *VHL* mutants in renal cancer. (**B**) Comparing *SETD2* cases to pan-negative (*VHL, PBRM1,* and *BAP1* negative) WT samples removes the confounding effect and finds 1664 differentially methylated CpGs, clustered using complete-linkage clustering and distance measure given by euclidean distance. (**C**) Genomic distribution of differentially methylated CpG sites shows that hypomethylated CpGs are strongly enriched in the gene bodies whereas hypermethylated CpGs show a more wide-spread distribution. Illumina array shows the distribution in the entire 450k methylation array; 3’UTR: 3’ untranslated region, 5’UTR: 5’ untranslated region; TSS1500: 1500 bp upstream of transcription start site, TSS200: 200 bp upstream of transcription start site (**D**) Gene enrichment analysis of differentially methylated sites. A large number of significantly enriched processes are involved in developmental pathways. The gene ratio refers to the number of genes in that gene set annotated to the GO term divided by the total number of genes in the gene set. A higher gene ratio implies greater overrepresentation of genes to the particular GO term.

When differentially methylated CpGs in a *VHL* mutant/*VHL* WT comparison are compared to differentially methylated CpGs between *SETD2* cases and *SETD2* controls, a large overlap can be seen for the differentially methylated CpG sites (Fig. 3A). This may confound the effect of *SETD2* variants on methylation. Therefore, to ensure that the methylation changes seen across renal cancers were not due to the confounding effect of *VHL* and other mutations, we selected a group of pan-negative WT samples by excluding samples with *VHL, BAP1,* or *PBRM1* mutations. We also excluded *VHL, BAP1*, and *PBRM1* mutants from the *SETD2* mutant cases to allow us to disentangle the effect of *SETD2* from other co-occuring mutations. 113 of the 259 *SETD2* control samples were also WT for *VHL, BAP1*, and *PBRM1* and the remaining 146 WT samples were excluded. When *SETD2* cases were compared to the pan-negative WT samples, 1664 CpGs were found to be differentially methylated (BH-adjusted p-value < 0.05). Even when the excluded samples were added back to the dataset, these 1664 CpGs performed better at unsupervised hierarchical clustering, giving a better separation of *SETD2* cases and controls compared to the differentially methylated CpGs found using the entire dataset (Fig. 3B). As in the previous analyses, the hypomethylated CpGs were enriched in gene bodies (Fig. 3C), providing further evidence that these SETD2-dependent methylation changes are due to the H3K36me3 axis. The top-most hypomethylated CpGs mapped to genes that lost H3K36me3 in SETD2 knock-outs or showed no change (representative figures in Supplementary Fig. 3). However, several of the most hypermethylated CpGs mapped to genes where H3K36me3 showed a redistribution from gene body to promoter regions although not all hypermethylated CpGs showed a distribution of H3K36me3 (Supplementary Fig. 4).

**Fig. 4:**
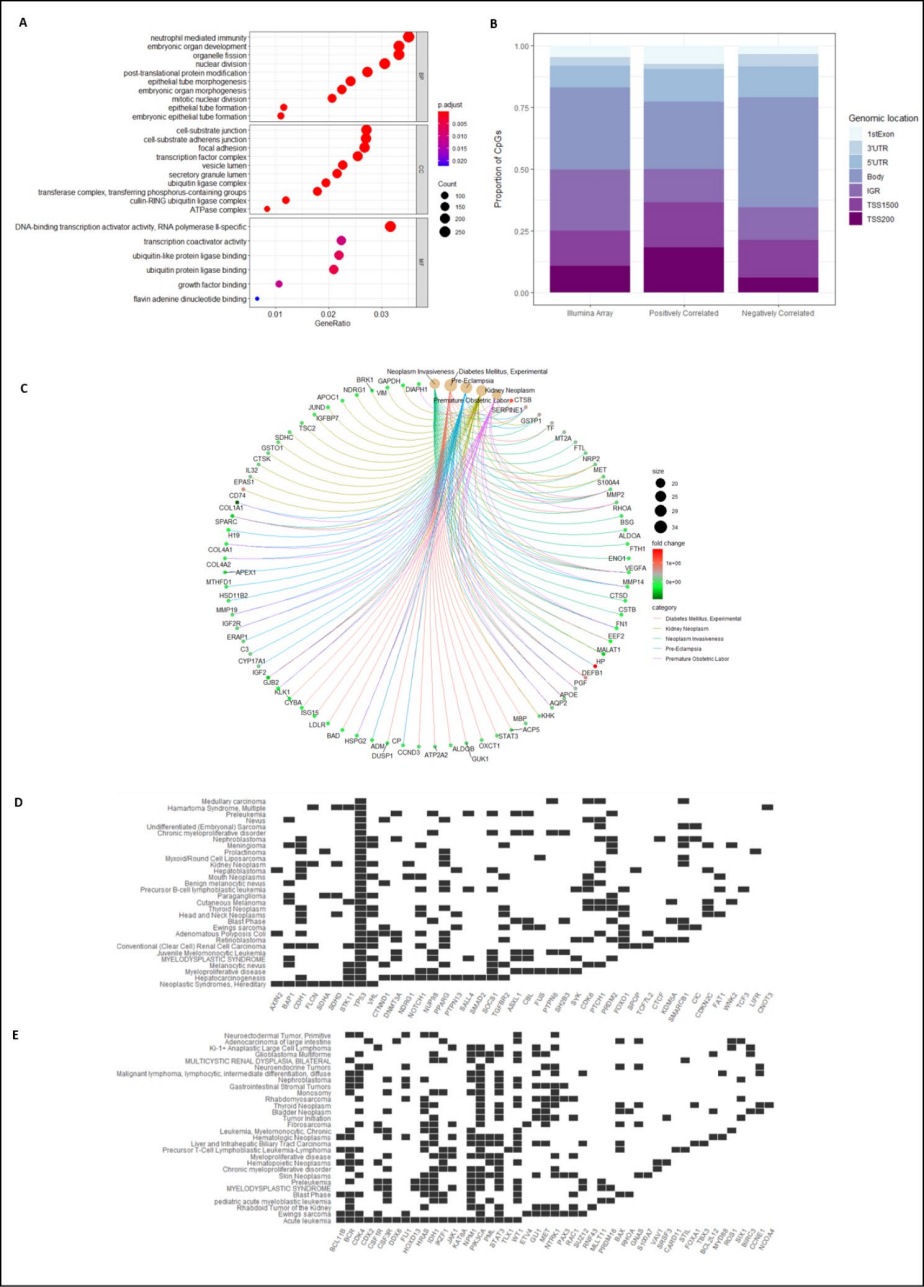
(**A**) shows the gene ontology results of genes whose expression and methylation are correlated. The gene ratio refers to the number of genes in that gene set annotated to the GO term divided by the total number of genes in the gene set. A higher gene ratio implies greater over-representation of genes to the particular GO term (**B**) Genes that were positively correlated with methylation were present more in transcription start sites or promoter regions whereas negatively correlated genes were more enriched in the gene bodies; 3’UTR: 3’ untranslated region, 5’UTR: 5’ untranslated region; TSS1500: 1500 bp upstream of transcription start site, TSS200: 200 bp upstream of transcription start site (**C**) Disease ontology of expression-methylation correlated genes shows that many of the differentially methylated genes are associated with kidney neoplasm and neoplasm invasiveness. (**D**) and (**E**) shows that a large number of these methylation-associated genes are tumour suppressor genes and oncogenes respectively.

A gene ontology analysis of the genes with differentially methylated CpGs revealed that the majority of genes were involved in developmental processes such as morphogenesis, embryonic organ development, and cell fate commitment (Fig. 3D). To explore whether these methylation changes had any impact on the expression levels of genes in these cancers, we conducted a methylation-gene expression correlation analysis. In order to characterise and categorise the function of the genes whose expression was found to be correlated with the methylation status of differentially methylated CpGs, we performed a gene ontology analysis based on biological processes, molecular functions and cellular components. Many of the genes were involved in embryonic development and morphogenesis (Fig. 4A). Interestingly, a large proportion of these genes were involved in immunity. Next, disease ontology and semantics analysis was performed to discover any disease associations of the genes that are correlated with differentially methylated CpGs. Figure 4 C shows the disease processes these genes are involved in. Genes whose expression was correlated with differential methylation in *SETD2* cases show an enrichment for processes such as neoplasm invasiveness, in particular kidney neoplasm, which suggests that *SETD2* mutations may be involved in cancer aggressiveness through DNA methylation. Interestingly, CpGs whose methylation was negatively correlated with gene expression were more enriched in gene bodies, whereas positively correlated CpGs had greater proportions in transcription start sites (Fig. 4B).

To test whether methylation changes had any impact on cancer genes, we examined which of the differentially methylated CpGs in *SETD2* cases were correlated with the expression of known cancer genes. Figure 4D and E show the tumour suppressor genes and oncogenes, respectively, whose expression is correlated with CpG sites that are differentially methylated in *SETD2* cases. Many of these are frequently mutated cancer genes including *TP53, FOXO1, CDK4*,and *PIK3CA* [39–42].

### A robust machine learning approach identifies a 3-CpG signature for the diagnosis of ***SETD2*** mutated cancers

33,153 differentially methylated CpGs were identified upon comparing *SETD2* mutated and *SETD2* control renal cancers (BH-adjusted p-value < 0.05), with 10,549 CpGs hypomethylated in *SETD2* cases and 22,604 CpGs hypermethylated CpGs. No difference was found in tumour purity or age (Welch Two Sample T-test, p > 0.05) nor in gender, race or ethnicity between *SETD2* mutants and *SETD2* WT samples (Pearson’s Chi-squared test, p > 0.05, Supplementary Table 1). Due to sample size constraints, in this analysis, we did not exclude *SETD2* mutants with VHL deletions. Additionally, since VHL deletions are likely to be present alongside *SETD2* deletions in the patient population, we decided to include these samples to accurately represent the patient population. Consistent with our predictions and observations in other cancers, CpGs that are hypomethylated in *SETD2* cases show strong enrichment in the gene bodies whereas hypermethylated CpGs do not.

To identify a generalisable CpG signature able to predict the *SETD2* status of samples, we used a multiple random sampling and cross-validation approach to perform CpG selection and misclassification error calculation at each training set size (see Materials and Methods). This allowed us to obtain a better estimation of the true error and thus generalisability of the selected model (more details in Materials and Methods).

**Fig. 5:**
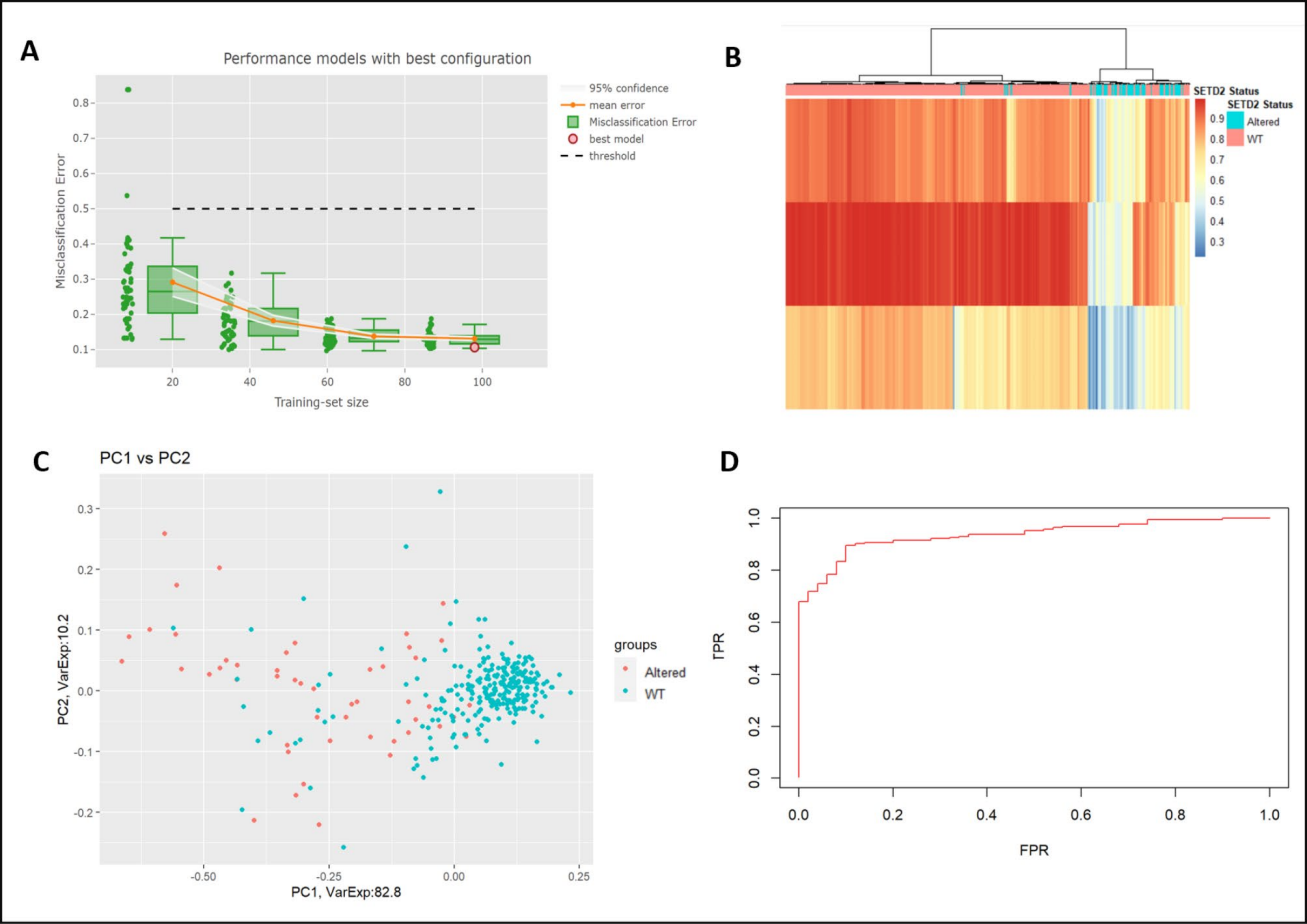
(**A**) Multiple random-sampling at different training set sizes shows that as the training-set size increases, the misclassification error greatly reduces. The interactive report shows the best model highlighted as the red circle at training set size of 98. (**B**) Heatmap displaying the methylation of the top 3 CpGs (cg14297023, cg17054691, and cg25415966) selected as the best model. Columns clustered using complete-linkage clustering and distance measure given by the Euclidean distance. (**C**) PCA analysis using the best model shows a good separation between *SETD2* cases and WT renal cancer samples. (**D**) ROC curve to display the true positive and false positive rates of the signature in classifying *SETD2* controls and cases.

Figure 5A shows that increasing the training set size reduces the misclassification error considerably, with a training-set size of 98 samples having the lowest error. Our approach yielded different “best models” based on various parameters of interest such as the model with the lowest mean-squared error or lowest absolute error. It was noted that two CpG sites (cg14297023 and cg25415966) were selected in all three of the best models based on misclassification error, mean-squared error, or mean absolute error.

Amongst the multiple iterations at a training set size of 98 samples, our best model was selected using the misclassification error and comprised only 3 CpG sites for prediction (Supplementary Table 3). These were cg14297023, cg17054691, and cg25415966, and could correctly identify renal cancer samples as *SETD2* cases or WT with a misclassification error of only 0.11. As there are multiple models at each training set size, we also calculated the probability measure of the likelihood of a certain CpG site being included in the various other models developed at a particular training set-size. This is given by the value p which shows that cg14297023 and cg25415966 have high probabilities and are, hence, more frequently selected in various best models of *SETD2* loss which suggests a stronger association with *SETD2* loss than other CpG sites (Supplementary Table 3). Figure 5B shows the methylation distribution of *SETD2* mutated and WT samples at each of the 3 CpG sites selected in the model. *SETD2* mutated samples on average have lower methylation at all three sites compared to WT samples. Next, we computed the principal components of the data considering only the 3 CpG sites and reported the PC1 vs. PC2 in a scatterplot (Fig. 5C). The first principal component, corresponding to 82.8% of variance, permits good separation of the two populations, i.e. *SETD2* mutated and WT, thus confirming the goodness of our model selection. Figure 5D shows a ROC curve of the best model with an area under the curve (ROC-AUC) of 0.94 which shows a very good prediction ability of the 3-CpG signature on the TCGA full set. As tumour staging may affect the incidence of *SETD2* mutations, we performed a chi-squared test to test the difference in tumour staging between *SETD2* control and *SETD2* case samples, which showed no significant difference (p = 0.373) (Supplementary Fig. 5).

**Table 3:**
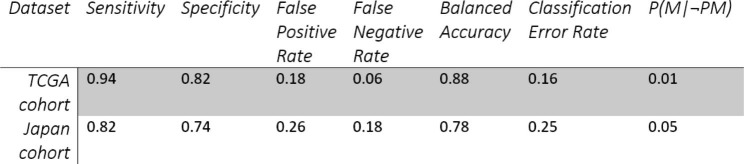
Model performance in TCGA and Japan cohort at a threshold of 0.66

We decided to investigate if the performance of our classifier could be improved by tuning the classification threshold of the binomial logistic regression model. We selected the threshold which maximized the Balanced Accuracy on the full training set, resulting in a cut-off of 0.66. This further improved the model, giving a sensitivity of 0.94, specificity of 0.82, and a false negative rate of 0.06 (Table 3).

**Fig. 6:**
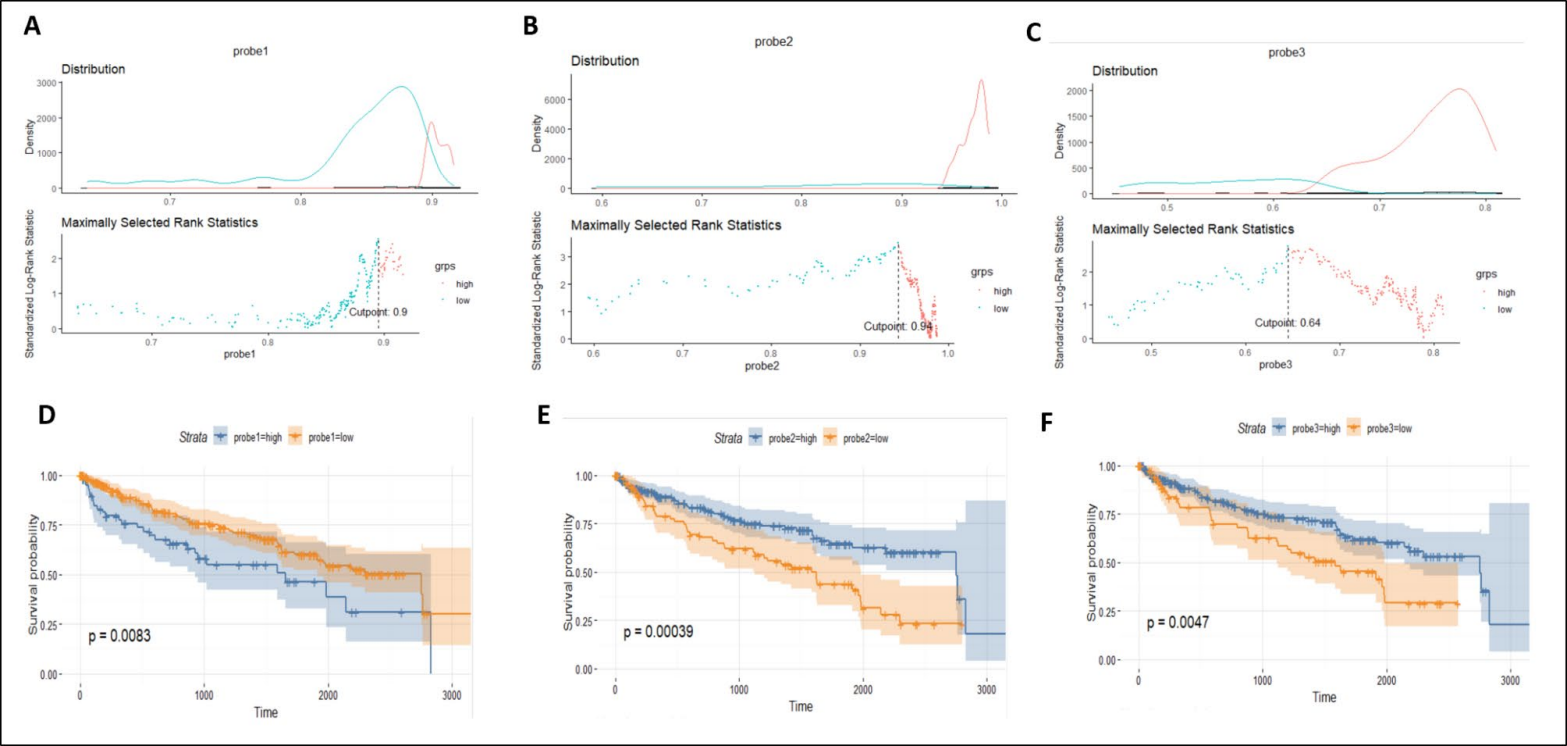
Panels **A-C** show the methylation distribution of each of the three CpGs- cg14297023, cg17054691, and cg25415966, respectively- with the maximally selected rank statistic which gives the greatest separation used to select the optimal cut-off for survival. Panels **D-F** show the Kaplan-Meier survival curve for each of the 3 CpGs. Each probe shows a significant difference in patient prognosis at the optimal cut-offs selected.

We also tested to see if the 3 CpGs could provide information on patient prognosis. We used the maximally selected rank statistic to determine the best cut-off for each probe (Fig. 6A-C). Figure 6(D-F) shows the Kaplan-Meier curves for each CpG site. There is a statistically significant difference in prognosis at a β-value cut-off of 0.90, 0.94, and 0.64 for cg14297023, cg17054691, and cg25415966 respectively. Supplementary Table 4 shows the methylation-expression correlation analyses for CpGs that are nearby these 3 CpGs. Our analysis found 14 CpGs hypomethylated in P4HB, 4 of which were associated with expression of the P4HB gene (Supplementary Table 4). Further, certain CpGs such as cg19936372 in P4HB are correlated to the expression of nearby genes as well as P4HB.

### Validation of our 3-CpG signature in an independent cohort

**Fig. 7:**
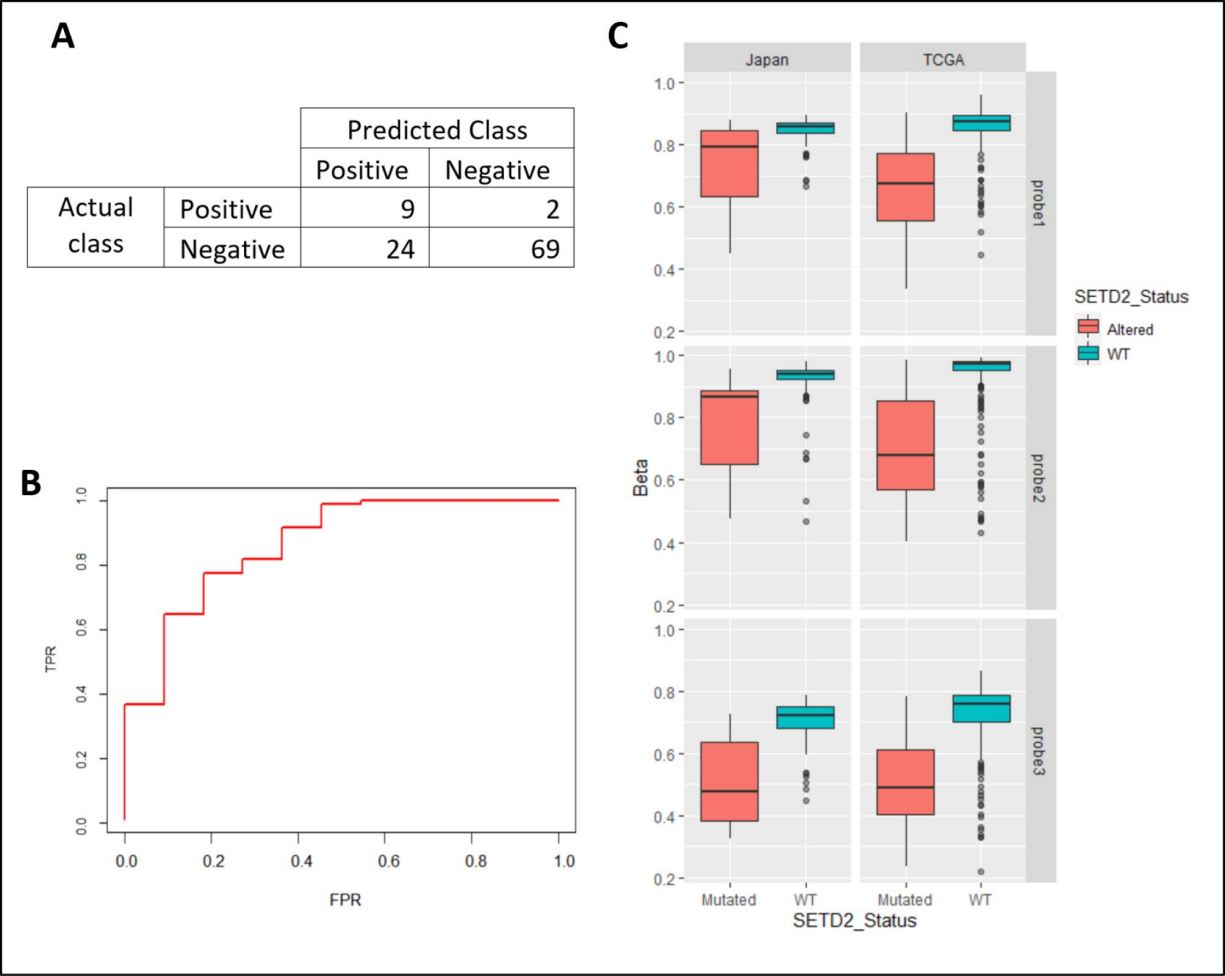
Validation of the biomarker in a Japanese cohort (**A**) shows that the 3-CpG signature shows an accuracy of 87% in classifying *SETD2* control and alternative variant cases. (**B**) shows the true positive and false positive rates of the signature in diagnosing *SETD2* control and altered samples in the Japanese cohort. (**C**) shows boxplots with the methylation distribution of each probe in both the Japanese and TCGA cohorts. Very similar distribution is seen in the *SETD2* altered and WT samples in each cohort. All three probes show hypomethylation in *SETD2* altered samples.

To test the performance of our model in an independent dataset, we used Illumina 450K array methylation data from a cohort of renal cancer patients in Japan [32]. The 3-CpG signature showed an accuracy of 87% in classifying *SETD2* control and alternative variant cases (Fig. 7A). Plotting an ROC curve showed that the 3 CpGs had an excellent performance in the Japanese cohort, with an AUC of 0.86 (Fig. 7B). The 3-CpG biomarker was able to correctly identify samples as *SETD2* cases or WT with a balanced accuracy of 0.78, the sensitivity of 0.82, and specificity of 0.74 (Table 3). The false-negative rate and the Bayes’ conditional probability of a sample being mutated given a classification as unmutated (P(M|¬PM)) were 0.18 and 0.05, respectively (Table 3). Figure 7C shows a side-by-side comparison of the methylation distribution of *SETD2* cases and *SETD2* control samples in the TCGA data as well as the Japanese cohort for each of the CpG sites in our biomarker. The methylation distribution was similar for both cohorts at each probe, with very similar medians and interquartile ranges. *SETD2* cases have reduced methylation at each of the 3 CpG sites compared to *SETD2* controls which is in line with our hypothesis of it being associated with loss of H3K36me3 at gene bodies.

## Discussion

### ***SETD2*** and DNA methylation

In this study, we conducted a pan-cancer analysis to disentangle DNA methylation changes associated with *SETD2* variants in cancer. We showed using tumour samples from the TCGA database that somatic *SETD2* variants, including single nucleotide variants and copy number variants are associated with widespread DNA methylation alterations across all cancer types tested. We also found that low *SETD2* expression in *SETD2* wild-type samples is also associated with DNA methylation changes. Previous studies on *SETD2* mutations and DNA methylation have highlighted the widespread promoter hypermethylation in *SETD2* mutants, which is in line with our results [21, 43]. Our study showed that a characteristic gene body hypomethylation phenotype is also present in both *SETD2* mutated and low *SETD2* expressing samples and is seen across multiple cancer types. Interestingly, these characteristic gene body hypomethylation changes can happen alongside the hypermethylation phenotype, which suggests that the methylation changes in two different directions may be due to two different mechanisms. This is also the first study to show the association between SETD2-dependent methylation dysregulation and gene expression in cancer, as well as its link to the tumorigenic process (discussed in more detail below). Thus, our study highlights a previously undescribed role of *SETD2* loss in tumorigenesis via DNA methylation dysregulation. This is also of particular clinical relevance as depletion of *SETD2* and H3K36me3 is associated with more aggressive tumours and worse prognosis [29–34].

In previous renal cancer studies, where *SETD2* mutations have been associated with DNA methylation changes, the effect of co-occuring mutations has not been accounted for [22, 37, 43, 44]. In renal cancer, *SETD2* mutations are frequently associated with *VHL, PBRM1*, and *BAP1* mutations as a result of chromosome 3p21 deletion [36]. All three mutations are also associated with DNA methylation changes which could potentially confound the association of *SETD2* with methylation [36, 38]. Similarly, studies on the CpG Island methylator phenotype (CIMP+) have highlighted the association of *SETD2* and DNA methylation in cancer, with gene body hypomethylation and promoter hypermethylation which is in line with our results [45–47]. While *SETD2* mutations can drive CIMP + phenotypes, not all CIMP + phenotypes occur due to *SETD2* mutations and Yates et al. found that *SETD2* was a driver of CIMP + in only 3 cancer types [46]. Further, several mutations may drive CIMP + in the same cancer type. For example, in mesothelioma, *KMT2B* is also a driver of CIMP alongside *SETD2*. Therefore, in order to disentangle *SETD2*-specific changes, we conducted differential methylation analysis specifically comparing *SETD2* WT and *SETD2* mutated groups to remove the effect of confounders and reveal the *SETD2*-specific methylation changes. Our study shows that the gene body hypomethylation can occur alongside CIMP + hypermethylation phenotype and are also seen in cancer types where *SETD2* is not a driver of CIMP + as well as those where it drives CIMP + phenotypes^46^.

Our results suggest that the DNA hypomethylation changes are likely due to a reduction of H3K6me3 upon *SETD2* loss or depletion, resulting in a reduced recruitment of DNMT3B through its PWWP domain and thus reduced methylation [18]. Both *SETD2* mutations, as well as reduced *SETD2* expression, are strongly associated with a reduction in H3K36me3 levels in cells [25, 33, 34]. We found a strong enrichment of hypomethylated CpGs in gene bodies, which is in line with this hypothesis as H3K36me3 is located primarily in gene body regions of transcribed genes [48]. This was further confirmed as the most hypomethylated CpGs in renal cancer identified in our study mapped to genes that lost H3K36me3 in *SETD2* knock-out renal carcinoma cell lines [25]. In terms of the strong hypermethylation phenotype occurring with *SETD2* mutation, this may be due to a redistribution of H3K36me3 to regions where it is not canonically present. This is highlighted in Tiedemann et al’s study which shows a marked redistribution of H3K36me3 from gene bodies to intergenic regions upon *SETD2* knock-out [43]. We also found that many of the top-most hypermethylated CpGs in renal cancer mapped to genes that showed a redistribution of H3K36me3 from gene bodies to promoters. This redistribution might also explain the CIMP hypermethylator phenotype associated with *SETD2* mutations [45–47].

### Role of DNA methylation in cancer pathogenesis and progression

Our methylation-expression correlation analysis shows that a large number of differentially methylated CpGs are associated with gene expression changes. It is interesting to note that a large proportion of CpGs whose methylation is negatively correlated with gene expression are present in the gene bodies which is unexpected given that gene body methylation is thought to be positively correlated with gene expression [49]. However, a few other studies have also found this negative correlation between gene body methylation and expression although the mechanism for this remains poorly understood [50–53]. Similarly, a fraction of genes showed a positive correlation between promoter hypermethylation and gene expression, which while contrary to the dogmatic understanding, is supported by other studies which have also observed a positive correlation between promoter methylation and gene expression, both in normal cells as well as in cancer contexts [54, 55]. The mechanism behind this has not been studied in detail, however, Wan et al’s findings suggest that there is enrichment of specific transcription factor motifs in positively correlated genes [55]. Other studies have also shown that some transcription factors preferentially bind to methylated DNA [56, 57]. This preferential recruitment of certain transcription factors to methylated promoters may be responsible for the increase in gene expression observed. Our gene ontology analyses show that a significant proportion of the genes whose expression was correlated with differentially methylated CpGs were enriched in various developmental processes. Cancer is a disease of dedifferentiation and reversal into more stem-like states, and the expression of many developmental genes is dysregulated in the tumorigenic process [58–60]. Our disease ontology analyses also showed that SETD2-dependent DNA methylation alterations were associated with altered expression of genes involved in kidney neoplasm and neoplasm invasiveness. Further, a large proportion of these genes are oncogenes or tumour suppressor genes such as *TP53, FOXO1, PIK3CA, and CDK4* [39–42]. Loss of SETD2 function is known to impact in kidney neoplasm formation through replication stress and impaired DNA repair [61, 62]. Our findings thus provide new insights into the functional impact of *SETD2* loss in cancer and suggest a new role for *SETD2* in tumorigenesis and cancer aggressiveness through DNA methylation dysregulation. Our choice of using a cut-off of 250,000 bp for the methylation-expression correlation analysis was in order to explore the long-range effects of SETD2-dependent methylation changes while minimizing the false positives. Kim et al. found that over 50% of the gene expression variation is explained through long-range methylation and can surprisingly be even more important than cis-methylation due to the higher order contacts between DNA as a result of the 3D architecture of the genome [63, 64]. While there is a benefit of using a more ‘targetted’ approach in reducing false positives, reducing the cut-off value comes with the risk of excluding potentially important CpGs that can provide new insights into the role of *SETD2* in cancer and the methylation-gene regulation axis, which was an important focus of our study.

### DNA methylation biomarker associated with ***SETD2*** mutation

Finally, we asked if we could apply our multiple random sampling and cross-validation approach to develop a DNA-methylation signature for *SETD2* cases, with the aim of developing a cheap clinical test for H3K36me3 depletion in future studies. Aware of the challenges in designing a good biomarker, we used robust machine learning to ensure robustness of our biomarker model. To make the biomarker more generalizable and avoid over-fitting, we used a repeated sampling approach with cross-validation (see Material and Methods). Our biomarker not only demonstrated an excellent performance in both TCGA data and the external cohort, but the model also performed very similarly in both populations with very close AUC and misclassification errors. Thus, this methodology allows for a better prediction of the true error of the model and its performance in the real world. This approach will enable us to select more generalizable and clinically-translatable biomarkers in the future.

All three of the genes mapped to the CpG loci in our signature have been previously shown to be associated with cancer. One of the probes in our 3-CpG signature, cg14297023, is located in the 3’ UTR of the *EIF3D* and regulates EIF3D expression [65]. Interestingly, EIF3D overexpression is a driver of sunitinib resistance in renal cancer and another study has shown that *SETD2* mutations are associated with sunitinib resistance [66, 67]. Cg17054691 is located in the *P4HB* gene, aberrant methylation and differential expression of which is implicated in the aetiology of prostate cancer [68]. Its overexpression is also linked to poorer survival in renal cancer as well as tumour progression in gliomas [69, 70]. Our analysis found 14 CpGs hypomethylated in *P4HB*, 4 of which were associated with expression of the *P4HB* gene. This is in line with our results above which suggest that SETD2-dependent methylation changes contribute to gene expression changes in cancer-associated genes, which could be a contributing factor in cancer etiology. While one CpG is unlikely to have an impact on gene expression, clusters of CpGs showing changes in the same direction more strongly correspond to gene expression changes [71]. Lastly, cg25415966 is located in *CABLES2* which shows differential methylation in cancer and was included in a DNA methylation-based prognostic biomarker in rectal cancer [72].

We have previously identified an evolutionarily conserved synthetic lethality between loss of *SETD2* and WEE1 inactivation, and that renal tumours with loss of *SETD2* or H3K36me3 can be specifically targeted with WEE1 inhibition, an observation that has been taken into clinical trials [24, 73, 74]. In current WEE1 trials, gene mutations are used to classify patients into WEE1-inhibition responders and non-responders [75]. However, in the case of *SETD2*, as the frequency of H3K36me3 is far greater than *SETD2* mutations a large proportion of patients that would benefit from WEE1 inhibition in an H3K36me3-loss background would be excluded in these trials. As H3K36me3 loss is linked to more aggressive tumours and worse prognosis, it is important to include such patients that would benefit from WEE1 inhibitor treatment [26, 31, 35, 76]. Thus, in the future, we would like to extend this study and validate the use of our biomarker in H3K36me3 depleted tumours with the aim of developing a DNA methylation blood-based biomarker. This would thus help select more patients that will benefit from WEE1 inhibitor or related treatments and would thus benefit far more patients. Our study serves as proof-of-principle that only a few CpGs can be used to develop a biomarker for *SETD2* loss and that a multiple resampling and cross-validation approach in the development of biomarkers provides a better estimate of the performance of biomarkers in independent datasets. We anticipate that increased use of this approach in future biomarker studies will lead to the selection of more generalizable models. We thus hope this will allow for the development of more clinically translatable biomarkers.

## Materials and methods

### Methylation data

Datasets for the pan-cancer methylation analysis were obtained from the cancer genome atlas (TCGA) [77]. Barcodes of samples that had *SETD2* mutation status and methylation data available were accessed using cBioPortal [78, 79]. Level 3 methylation data from Infinium HumanMethylation450 BeadChip was downloaded from the TCGA harmonized datasets, using TCGA-biolinks in R/Bioconductor [80–83]. The methylation at each probe is given by β-values for each CpG, calculated as M/(M + U), where M is the signal intensity at the methylated bead and U is the signal intensity at the unmethylated bead. Genomic coordinates of the CpGs were mapped according to the GRCh38 build of the reference genome. For cancer types that had lower than 3% *SETD2* mutation frequency or fewer than 15 *SETD2* case samples in the TCGA study, *SETD2* expression in the form of mRNA RSEM values were downloaded from cBioPortal. Samples marked as mutated include all alterations including missense mutations, splice mutations, truncating mutations, as well as copy number variations such as deep deletions. Samples marked as WT have no alterations. A large proportion of copy number variations in renal cancer were homozygous deletions. WT are samples with no alterations. Samples in the top and bottom quartile of *SETD2* expression based on mRNA RSEM values were analysed for the effect of *SETD2* expression on DNA methylation.

### Differential methylation analysis

For the DNA methylation analysis, probes with NA values were removed. Samples with *SETD2* amplifications were excluded to remove the confounding effect of H3K36me3 overexpression. Differential methylation analysis between *SETD2* cases and *SETD2* control samples was performed using the champ. DMP function in ChAMP Bioconductor on R Studio [84]. The limma method was used to identify differentially methylated CpGs [85, 86]. CpGs with an adjusted p-value cut-off < 0.05 were considered significant. The Benjamini-Hochberg method was used to correct for multiple testing [87]. Genomic distribution analysis of differentially methylated CpGs was performed using the Shiny Library within ChAMP for gene body enrichment of differentially methylated CpGs [88]. For TCGA-KIRC, the *SETD2* mutations details for samples are added in supplementary Tables 5 to enhance replication.

### Gene ontology analyses

Differentially methylated CpGs were mapped onto corresponding genes using ChAMP. Duplicate genes were removed and the Gene Ontology over-representation test performed using the enrichGO function in clusterProfiler (version 3.14.3) with an adjusted p-value cutoff of < 0.05 and multiple testing correction applied using the Benjamini-Hochberg method [89, 90]. Gene ontology analyses were performed using three ontologies – molecular function (MF), cellular component (CC), and biological process (BP).

### Methylation-expression correlation analysis

To determine which differentially methylated CpGs were correlated with expression of neighbouring genes, a methylation-expression correlation analysis was performed using the MEAL, minfi, and missMethyl packages [91–93]. The BiomaRt package was used to access genomic coordinates and gene annotations from the ensemble database [94–96]. For each CpG probe, a flanking region of 250,000 base pairs was selected and correlation between a CpG and any genes in its flanking region determined using the correlationMethExprs function in the MEAL package [93]. Pairs with a BH-adjusted p-value < 0.05 were selected as significantly correlated.

### Disease ontology and semantic analyses

DOSE (version 3.12.0) was used to perform disease ontology and semantic analyses of methylation-expression correlated genes in renal cancer to discover disease associations of genes that showed a correlation with differentially methylated CpGs [97]. A hypergeometric model is implemented within DOSE. The minimum gene size for testing was set to 10 and the maximum to 500 and a Benjamini-Hochberg adjusted p-value cut-off of 0.05 was selected to determine significant disease associations.

### Visualization of enrichment analyses

The enrichment results for the gene ontology and disease ontology analyses generated using clusterProfiler and DOSE were visualized using the enrichplot package (version 1.6.1) [98]. The barplots and dotplots were created using the barplot and dotplot functions respectively. The gene concept networks in Fig. 4B were created using cnetplot function. The oncogene and tumour suppressor heatmap in Fig. 4D and E was created using the heatplot function.

### Development of methylation signature for ***SETD2*** loss in renal cancer

To develop the DNA methylation biomarker for renal cancer, Level 3 methylation data from the Infinium HumanMethylation450 BeadChip was downloaded for the TCGA-KIRC harmonized dataset [22]. In total, 309 samples had DNA methylation data available, of which 50 were *SETD2* cases. Probes with NA values in more than 50% of the samples were removed and the top quartile of most variable probes were selected as a pre-processing step.

To study the relationship between methylation data and *SETD2* status we used a robust approach based on features screening and selection followed by generalised linear model selection with L1/L2 penalisation, with hyperparameters optimised in 10-fold cross-validation. The featurescreening step was used to pre-select the features most correlated with the outcome and performed through an empirical Bayes moderated t-statistics test. We considered three hyperparameters: the number of features to keep in the screening, the alpha and lambda parameters in penalised models. To obtain a better estimation of the true error of the methodology, a multiple random-sampling approach spanning different training-set sizes was adopted. For each size, data was randomly split into training and test sets: the training data was used to fit the model, while the test set was used to assess its performance. The hyperparameters of the models were selected from a grid of provided values via 10-fold cross-validation in order to obtain the minimum mean cross-validated error. We used the binomial deviance as a measure of accuracy. After the hyperparameters were fixed, the final model was fitted on the entire training set and tested on the left-out data. The above steps were repeated for multiple random samples of the data, in order to estimate the mean error of our procedure and the related 95% confidence interval (CI). The best model was selected as the model with the minimum test error across all the models fitted using the training-set size showing the lowest upper bound of the 95% CI. ROC curves and PCA plots were plotted. As our binomial logistic regression model returns a probability, the conversion to a class label is obtained via the definition of a classification threshold (which has a default value of 0.5) so that all values equal to or greater than the cut-off are mapped to one class, and all the remaining values are mapped to the other class. Best model was tuned by selecting the best cut-off as threshold.

To calculate the probability of a sample being true *SETD2* mutant when predicted as mutant (true positive, $P\left(M\right|PM)$) or WT (false negative, or missed *SETD2* cases, $P\left(M\right|\neg PM)$), we used the Bayes theorem.

Probability of *SETD2* mutation when predicted to be *SETD2* mutant was calculated as:

$$P\left(M\right|PM) =$$$$\frac{P\left(PM\right|M)\cdot P(M)}{P\left(PM\right)}$$, where $$M$$ means *SETD2* mutant, $$PM$$ and $$\neg PM$$ are predicted *SETD2* case and WT respectively, and $$P\left(M\right)$$

is the prior probability, i.e. probability of having *SETD2* case among all cancer cases (estimated from training dataset from TCGA), $$P\left(PM\right|M)$$ is a likelihood of classifier predicting *SETD2* case if the sample is actually *SETD2* case. Thus, $$P\left(PM\right|M)$$ is the true positive rate of classification. Finally, $$P\left(PM\right)= P\left(PM\right|M)$$$$\cdot$$$$P\left(M\right) + P\left(PM\right|\neg M)$$$$\cdot$$$$P(\neg M)$$ is a marginal probability, where $$P(\neg M)$$ is a ratio of *SETD2* among all cancer cases (calculated simply as $$P(\neg M) = 1-P\left(M\right)$$); $$P\left(PM\right|\neg M)$$ is a probability of classifier predicting *SETD2* case status to a sample actually of *SETD2* control (false positive rate).

Probability of actual *SETD2* mutant sample if it is predicted to be *SETD2* WT is:

$$P\left(M\right|\neg PM) =$$$$\frac{P(\neg PM|M)\cdot P(M)}{P(\neg PM)}$$where $$P(\neg PM|M)$$ is a likelihood of classifier predicting *SETD2* WT if the sample is actually a *SETD2* mutant. $$P(\neg PM|M)$$ is simply false negative rate of classification. Finally, $$P(\neg PM)= P(\neg PM|M)$$$$\cdot$$$$P\left(M\right) + P(\neg PM|\neg M)$$$$\cdot$$$$P(\neg M)$$ is a marginal probability, where $$P(\neg PM|\neg M)$$ is a probability of classifier predicting *SETD2* control status to a sample actually of *SETD2* control (simply true negative rate).

For independent validation of our model, methylation data from the Infinium HumanMethylation450 BeadChip and whole genome sequencing data were accessed for a cohort of clear cell renal cell carcinoma patients in Japan [32]. The best model was tested on the Japanese dataset. Dataset characteristics for the Japanese cohort have been detailed previously and the data can be accessed on the European Genome-phenome Archive (EGA) under EGAS00001000509.

### Survival analysis

Survival data for the TCGA-KIRC renal cancer dataset was downloaded using RTCGA.clinical package (version 20151101.16.0) [99]. The maximally selected LogRank statistic was calculated for each probe using the maxstat package in order to find the optimal β-value cut-off that gave a statistically significant association with prognosis [100, 101]. Survival analyses were then performed using this cut-off by the Kaplan-Meier method using the package survival (version 3.2–11) [102, 103]. Kaplan-Meier curves were generated using survminer (version 0.4.9) [99, 104].

### Plots

Venn diagrams were generated using the VennDiagram package, heatmaps to visualize differentially methylated CpGs were generated using pheatmap package, and boxplots were generated using ggplot2 [105–107].

## Electronic supplementary material

Below is the link to the electronic supplementary material.

Supplementary Material 1

## Data Availability

All TCGA datasets are publicly available. Data from the Japanese cohort are deposited in the European Genome-phenome Archive (EGA) under accession EGAS00001000509 and available under request [32]. H3K36me3 Chip-Seq data in SETD2 WT and KO renal cancer cell lines are available under GEO accession GSE66884 [25].

## Declarations

### Materials and correspondence

Materials and correspondence to be addressed to T.C.H.

## References

[1] Moore LD, Le T, Fan G (2013). DNA methylation and its basic function. Neuropsychopharmacology.

[2] Brenet F et al. DNA methylation of the First exon is tightly linked to transcriptional silencing. PLoS ONE 6, (2011). 10.1371/journal.pone.0014524 PMC3022582 21267076

[3] Hellman A, Chess A (2007). Gene body-specific methylation on the active X chromosome. Sci (80-).

[4] Haffner MC (2011). Global 5-hydroxymethylcytosine content is significantly reduced in tissue stem/progenitor cell compartments and in human cancers. Oncotarget.

[5] Nishiyama A, Nakanishi M (2021). Navigating the DNA methylation landscape of cancer. Trends Genet.

[6] Wajed SA, Laird PW, DeMeester TR (2001). DNA methylation: an alternative pathway to Cancer. Ann Surg.

[7] Hu X et al. Evolution of DNA methylome from precancerous lesions to invasive lung adenocarcinomas. *Nat. Commun* 2021 121 12, 1–13 (2021). 10.1038/s41467-021-20907-z PMC7846738 33514726

[8] Wajed SA, Laird PW, DeMeester TR. *No title*. *Annals of surgery*. Volume 234. Lippincott, Williams, and Wilkins; 2001. 10.1097/00000658-200107000-00003 PMC1421942 11420478

[9] DONG Y, ZHAO H, LI H, LI X, YANG (2014). DNA methylation as an early diagnostic marker of cancer (review). Biomed Rep.

[10] Flavahan WA, Gaskell E, Bernstein BE. Epigenetic plasticity and the hallmarks of cancer. Science 357, (2017). 10.1126/science.aal2380 PMC5940341 28729483

[11] Locke WJ (2019). DNA methylation Cancer biomarkers: translation to the clinic. Front Genet.

[12] Mikeska T, Bock C, Do H, Dobrovic A. DNA methylation biomarkers in cancer: progress towards clinical implementation. 10.1586/erm.12.45 2014;12:473–87 10.1586/erm.12.45 22702364

[13] Mayeux R (2004). Biomarkers: potential Uses and Limitations. NeuroRx.

[14] Mikeska T, Craig JM (2014). DNA methylation biomarkers: Cancer and Beyond. Genes (Basel).

[15] Drucker E, Krapfenbauer K (2013). Pitfalls and limitations in translation from biomarker discovery to clinical utility in predictive and personalised medicine. EPMA J.

[16] Du J, Johnson LM, Jacobsen SE, Patel DJ. DNA methylation pathways and their crosstalk with histone methylation. *Nat. Rev. Mol. Cell Biol* 2015 169 16, 519–532 (2015). 10.1038/nrm4043 PMC4672940 26296162

[17] Baubec T (2015). Genomic profiling of DNA methyltransferases reveals a role for DNMT3B in genic methylation. Nature.

[18] Rondelet G, Maso D, Willems T, Wouters J (2016). Structural basis for recognition of histone H3K36me3 nucleosome by human de novo DNA methyltransferases 3A and 3B. J Struct Biol.

[19] Jeziorska DM (2017). DNA methylation of intragenic CpG islands depends on their transcriptional activity during differentiation and disease. Proc Natl Acad Sci U S A.

[20] Neri F et al. Intragenic DNA methylation prevents spurious transcription initiation. 543, 72–7 (2017). 10.1038/nature21373 28225755

[21] Hahn MA, Wu X, Li AX, Hahn T, Pfeifer GP (2011). Relationship between gene body DNA methylation and intragenic H3K9me3 and H3K36me3 chromatin Marks. PLoS ONE.

[22] Creighton CJ (2013). Comprehensive molecular characterization of clear cell renal cell carcinoma. Nature.

[23] Gerlinger M (2014). Genomic architecture and evolution of clear cell renal cell carcinomas defined by multiregion sequencing. Nat Genet.

[24] Pfister SX (2015). Inhibiting WEE1 selectively kills histone H3K36me3-Deficient cancers by dNTP starvation. Cancer Cell.

[25] Ho TH (2015). High-resolution profiling of histone h3 lysine 36 trimethylation in metastatic renal cell carcinoma. Oncogene 2016 3512.

[26] Fontebasso AM (2013). Mutations in SETD2 and genes affecting histone H3K36 methylation target hemispheric high-grade gliomas. Acta Neuropathol.

[27] Tsang JY et al. SETD2 alterations and histone H3K36 trimethylation in phyllodes tumor of breast. *Breast Cancer Res. Treat* 2021 1872 187, 339–347 (2021). 10.1007/s10549-021-06181-z 33844099

[28] Ettel M, Zhao L, Schechter S, Shi J (2019). Expression and prognostic value of NSD1 and SETD2 in pancreatic ductal adenocarcinoma and its precursor lesions. Pathology.

[29] La Rochelle J (2010). Chromosome 9p deletions identify an aggressive phenotype of clear cell renal cell carcinoma. Cancer.

[30] Ho TH et al. Loss of histone H3 lysine 36 trimethylation is associated with an increased risk of renal cell carcinoma-specific death. *Mod. Pathol* 2016 291 29, 34–42 (2015). 10.1038/modpathol.2015.123 PMC4697879 26516698

[31] Hakimi AA (2013). Adverse outcomes in Clear Cell Renal Cell Carcinoma with mutations of 3p21 epigenetic regulators BAP1 and SETD2: a report by MSKCC and the KIRC TCGA Research Network. Clin Cancer Res.

[32] Sato Y et al. Integrated molecular analysis of clear-cell renal cell carcinoma. *Nat. Genet* 2013 458 45, 860–867 (2013). 10.1038/ng.2699 23797736

[33] Liu W et al. Decreased Expression of SETD2 Predicts Unfavorable Prognosis in Patients With Nonmetastatic Clear-Cell Renal Cell Carcinoma. *Medicine (Baltimore)* 94, e2004 (2015). 10.1097/MD.0000000000002004 PMC4912287 26559293

[34] Pecce V (2020). Loss of function SETD2 mutations in poorly differentiated Metastases from two Hürthle Cell Carcinomas of the thyroid. Cancers (Basel).

[35] Huang KK (2016). SETD2 histone modifier loss in aggressive GI stromal tumours. Gut.

[36] Robinson CM (2018). Consequences of VHL loss on global DNA methylome. Sci Rep.

[37] Ricketts CJ et al. The Cancer Genome Atlas Comprehensive Molecular characterization of renal cell carcinoma. Cell Rep 23, (2018). 10.1016/j.celrep.2018.03.075 PMC6075733 29617669

[38] Wang Y, Guo X, Bray MJ, Ding Z, Zhao Z. An integrative genomics approach for identifying novel functional consequences of PBRM1 truncated mutations in clear cell renal cell carcinoma (ccRCC). 17, 227–37 (2016). 10.1186/s12864-016-2906-9 PMC5001239 27556922

[39] Olivier M, Hollstein M, Hainaut P. TP53 mutations in human cancers: origins, consequences, and clinical use. Cold Spring Harb Perspect Biol 2, (2010). 10.1101/cshperspect.a001008 PMC2827900 20182602

[40] Zhang B, Gui LS, Zhao XL, Zhu LL, Li (2015). Q. W. FOXO1 is a tumor suppressor in cervical cancer. Genet Mol Res.

[41] Baker SJ, Reddy EP (2012). CDK4: a key player in the cell cycle, Development, and Cancer. Genes Cancer.

[42] Samuels Y, Waldman T (2010). Oncogenic mutations of PIK3CA in human cancers. Curr Top Microbiol Immunol.

[43] Tiedemann RL (2016). Dynamic reprogramming of DNA methylation in SETD2-deregulated renal cell carcinoma. Oncotarget.

[44] Chen YC, Gotea V, Margolin G, Elnitski L. Significant associations between driver gene mutations and DNA methylation alterations across many cancer types. PLoS Comput Biol 13, (2017). 10.1371/journal.pcbi.1005840 PMC5709060 29125844

[45] Sánchez-Vega F, Gotea V, Margolin G, Elnitski L (2015). Pan-cancer stratification of solid human epithelial tumors and cancer cell lines reveals commonalities and tissue-specific features of the CpG island methylator phenotype. Epigenetics and Chromatin.

[46] Yates J, Boeva V (2022). Deciphering the etiology and role in oncogenic transformation of the CpG island methylator phenotype: a pan-cancer analysis. Brief Bioinform.

[47] Saghafinia S, Mina M, Riggi N, Hanahan D, Ciriello G. Pan-Cancer Landscape of aberrant DNA methylation across human tumors. Cell Rep 25, (2018). 10.1016/j.celrep.2018.09.082 30355485

[48] KO K (2005). A novel domain in Set2 mediates RNA polymerase II interaction and couples histone H3 K36 methylation with transcript elongation. Mol Cell Biol.

[49] Ball MP (2009). Targeted and genome-scale methylomics reveals gene body signatures in human cell lines. Nat Biotechnol.

[50] Arechederra M (2018). Hypermethylation of gene body CpG islands predicts high dosage of functional oncogenes in liver cancer. Nat Commun.

[51] Lim YC (2017). A complex association between DNA methylation and gene expression in human placenta at first and third trimesters. PLoS ONE.

[52] Spainhour JC, Lim HS, Yi SV, Qiu P. Correlation patterns between DNA methylation and gene expression in the Cancer Genome Atlas. Cancer Inf 18, (2019). 10.1177/1176935119828776 PMC6376553 30792573

[53] Yang X (2014). Gene body methylation can alter Gene expression and is a therapeutic target in Cancer. Cancer Cell.

[54] Rauluseviciute I, Drabløs F, Rye MB. DNA hypermethylation associated with upregulated gene expression in prostate cancer demonstrates the diversity of epigenetic regulation. BMC Med Genomics 13, (2020). 10.1186/s12920-020-0657-6 PMC6950795 31914996

[55] Wan J et al. Characterization of tissue-specific differential DNA methylation suggests distinct modes of positive and negative gene expression regulation. BMC Genomics 16, (2015). 10.1186/s12864-015-1271-4 PMC4331481 25652663

[56] Hu S et al. DNA methylation presents distinct binding sites for human transcription factors. *Elife* 2013, (2013). 10.7554/eLife.00726 PMC3762332 24015356

[57] Zhu H, Wang G, Qian J. Transcription factors as readers and effectors of DNA methylation. Nat Rev Genet 17, (2016). 10.1038/nrg.2016.83 PMC5559737 27479905

[58] Aiello NM, Stanger BZ (2016). Echoes of the embryo: using the developmental biology toolkit to study cancer. Dis Model Mech.

[59] Kho AT (2004). Conserved mechanisms across development and tumorigenesis revealed by a mouse development perspective of human cancers. Genes Dev.

[60] Naxerova K (2008). Analysis of gene expression in a developmental context emphasizes distinct biological leitmotifs in human cancers. Genome Biol 2008.

[61] Pfister SX (2014). SETD2-Dependent histone H3K36 trimethylation is required for homologous recombination repair and Genome Stability. Cell Rep.

[62] Kanu N (2015). SETD2 loss-of-function promotes renal cancer branched evolution through replication stress and impaired DNA repair. Oncogene 2015 3446.

[63] Kim S, Park HJ, Cui X, Zhi D (2020). Collective effects of long-range DNA methylations predict gene expressions and estimate phenotypes in cancer. Sci Rep.

[64] Beagrie RA et al. Complex multi-enhancer contacts captured by genome architecture mapping. Nature 543, (2017). 10.1038/nature21411 PMC5366070 28273065

[65] Wang D, Jia Y, Zheng W, Li C, Cui W. Overexpression of eIF3D in lung adenocarcinoma is a new independent prognostic marker of poor survival. Dis Markers (2019). 10.1155/2019/6019637 PMC6925810 31885740

[66] Elgendy M (2019). Identification of mutations associated with acquired resistance to sunitinib in renal cell cancer. Int J Cancer.

[67] Huang H (2019). EIF3D promotes sunitinib resistance of renal cell carcinoma by interacting with GRP78 and inhibiting its degradation. EBioMedicine.

[68] Wang L, Wang B, Quan Z (2020). Identification of aberrantly methylated-differentially expressed genes and gene ontology in prostate cancer. Mol Med Rep.

[69] Zhu Z (2019). Overexpression of P4HB is correlated with poor prognosis in human clear cell renal cell carcinoma. Cancer Biomarkers.

[70] Zou H (2018). P4HB and PDIA3 are associated with tumor progression and therapeutic outcome of diffuse gliomas. Oncol Rep.

[71] Jaffe AE (2012). Bump hunting to identify differentially methylated regions in epigenetic epidemiology studies. Int J Epidemiol.

[72] Gaedcke J (2014). Identification of a DNA methylation signature to predict disease-free survival in locally advanced rectal cancer. Oncotarget.

[73] National Cancer Institute. (2017, S.-). *Testing AZD1775 in Advanced Solid Tumors That Have a Mutation Called SETD2. ClinicalTrials.gov Identifier: NCT03284385*. https://clinicaltrials.gov/ct2/show/NCT03284385

[74] Pai CC et al. An essential role for dNTP homeostasis following CDK-induced replication stress. J Cell Sci 132, (2019). 10.1242/jcs.226969 PMC6451416 30674555

[75] Seligmann JF (2021). Inhibition of WEE1 is effective in TP53- and RAS-Mutant metastatic colorectal Cancer: a Randomized Trial (FOCUS4-C) comparing Adavosertib (AZD1775) with active monitoring. J Clin Oncol.

[76] Sarakbi W, Al (2009). The mRNA expression of SETD2 in human breast cancer: correlation with clinico-pathological parameters. BMC Cancer.

[77] Hudson TJ (2010). International network of cancer genome projects. Nat 2010 4647291.

[78] Cerami E (2012). The cBio Cancer Genomics Portal: an Open platform for exploring Multidimensional Cancer Genomics Data. Cancer Discov.

[79] Gao J (2013). Integrative analysis of Complex Cancer Genomics and Clinical Profiles using the cBioPortal. Sci Signal.

[80] Colaprico A (2016). TCGAbiolinks: an R/Bioconductor package for integrative analysis of TCGA data. Nucleic Acids Res.

[81] Huber W (2015). Orchestrating high-throughput genomic analysis with Bioconductor. Nat Methods.

[82] Mounir M (2019). New functionalities in the TCGAbiolinks package for the study and integration of cancer data from GDC and GTEx. PLOS Comput Biol.

[83] Silva TC et al. TCGA Workflow: Analyze cancer genomics and epigenomics data using Bioconductor packages. *F1000Research* 5, (2016). 10.12688/f1000research.8923.2 PMC5302158 28232861

[84] Morris TJ et al. ChAMP: 450k chip analysis methylation Pipeline. Bioinformatics 30, (2014). 10.1093/bioinformatics/btt684 PMC3904520 24336642

[85] Wettenhall JM, Smyth GK, limmaGUI (2004). A graphical user interface for linear modeling of microarray data. Bioinformatics.

[86] Smyth GK. Linear Models and empirical Bayes methods for assessing Differential expression in microarray experiments. Stat Appl Genet Mol Biol 3, (2004). 10.2202/1544-6115.1027 16646809

[87] Benjamini Y, Hochberg Y (1995). Controlling the false Discovery rate: a practical and powerful Approach to multiple testing. J R Stat Soc Ser B.

[88] Chang W et al. Shiny: web application Framework for R. R package version 0.9.1. https://cran.r-project.org/package=shiny (2021).

[89] Wu T, et al. Journal Pre-proof clusterProfiler 4.0: a universal enrichment tool for interpreting omics data. Innov. 2021;100141. 10.1016/j.xinn.2021.100141 10.1016/j.xinn.2021.100141 PMC8454663 34557778

[90] Yu G, Wang L-G, Han Y, He Q-Y. clusterProfiler: an R Package for Comparing Biological Themes Among Gene Clusters. https://home.liebertpub.com/omi 2012;16:284–287 10.1089/omi.2011.0118 PMC3339379 22455463

[91] MJ A (2014). Minfi: a flexible and comprehensive Bioconductor package for the analysis of Infinium DNA methylation microarrays. Bioinformatics.

[92] Phipson B, Maksimovic J, Oshlack A (2016). missMethyl: an R package for analyzing data from Illumina’s HumanMethylation450 platform. Bioinformatics.

[93] Ruiz-Arenas C, Gonzalez JR. MEAL: Perform methylation analysis. (2021).

[94] Durinck S, Spellman PT, Birney E, Huber W (2009). Mapping identifiers for the integration of genomic datasets with the R/Bioconductor package biomaRt. Nat Protoc.

[95] S D (2005). BioMart and Bioconductor: a powerful link between biological databases and microarray data analysis. Bioinformatics.

[96] Yates AD (2020). Ensembl 2020. Nucleic Acids Res.

[97] Yu G, Wang L-G, Yan G-R, He Q-Y (2015). DOSE: an R/Bioconductor package for disease ontology semantic and enrichment analysis. Bioinformatics.

[98] G Y, enrichplot. Visualization of Functional Enrichment Result. R package version 1.12.2. (2021).

[99] M K. RTCGA.clinical: Clinical datasets from The Cancer Genome Atlas Project. R package version 20151101.22.0. (2021).

[100] Hothorn T. Maximally Selected Rank Statistics. R package version 0.7–25. (2017).

[101] Lausen B, Schumacher M (1992). Maximally Sel Rank Stat Biometrics.

[102] Kaplan EL, Meier P (1958). Nonparametric estimation from incomplete observations. J Am Stat Assoc.

[103] T TA. Package for Survival Analysis in R. (2021).

[104] Kassambara A, Kosinski M, Biecek P. Drawing Survival Curves using ‘ggplot2’. R package version 0.4.9. (2021).

[105] Chen H, VennDiagram. Generate High-Resolution Venn and Euler Plots. R package version 1.6.20. (2018).

[106] Kolde R, pheatmap. Pretty Heatmaps. R package version 1.0.12. (2019).

[107] Wickham H (2016). ggplot2: elegant graphics for data analysis.

