# Supplementary material for: A role for SETD2 loss in tumorigenesis through DNA methylation dysregulation
Source: BMC Cancer. 2023 Aug 1;23:721. doi: 10.1186/s12885-023-11162-0 (PMC10394884; doi:10.1186/s12885-023-11162-0)

**Supplementary Table 1. cohort characteristics for TCGA-KIRC dataset**

|  | **All samples** | **SETD2 Mutant** | **SETD2 WT** |
| --- | --- | --- | --- |
| **n** | 309 | 60 | 259 |
| **Sex, n** |  |  |  |
| **Female** | 112 | 16 | 96 |
| **Male** | 197 | 34 | 163 |
| **Age, mean (SD), years** | 61.45 (11.68) | 60.52 (9.89) | 61.63 (12.00) |
| **Race/Ethnicity, n** |  |  |  |
| **White** | 257 | 42 | 215 |
| **Black/African American** | 48 | 6 | 42 |
| **Asian** | 1 | 1 | 0 |
| **NA** | 3 | 1 | 2 |
| **Stage, n** |  |  |  |
| **T1** | 151 | 12 | 136 |
| **T2** | 40 | 6 | 34 |
| **T3** | 110 | 28 | 82 |
| **T4** | 8 | 1 | 7 |
| **Tumour Purity, mean (SD)** | 0.67 (0.13) | 0.65 (0.14) | 0.67 (0.13) |
| **Radiation Therapy, n** |  |  |  |
| **Yes** | 2 | 2 | 0 |
| **No** | 113 | 18 | 95 |
| **NA** | 194 | 30 | 164 |
| **Overall Survival, mean (SD), days** | 1366 (1058) | 1324 (1052) | 1581 (1071) |
|  |  |  |  |
| Note that: 1 missing value in tumour purity measures, 12 missing values in Overall Survival data |  |  |  |

**Supplementary Table 2. SETD2 mutations co-occur with BAP1, VHL, and PBRM1 in renal cancer**


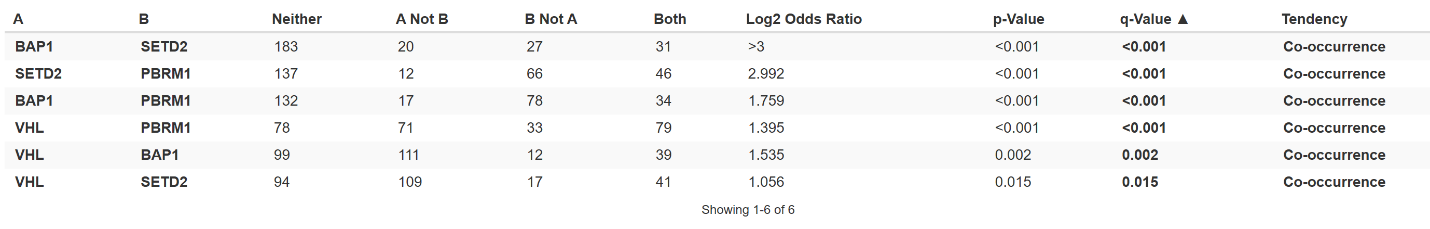


**Supplementary Table 3: Details on CpG sites selected in the best model; p is the number of times the feature was recruited across all the models obtained via resampling for the training set size divided by the number of repeats; score_t and score_w are feature importance score calculated considering the accuracy measures computed over the testing set and whole data set, respectively.**


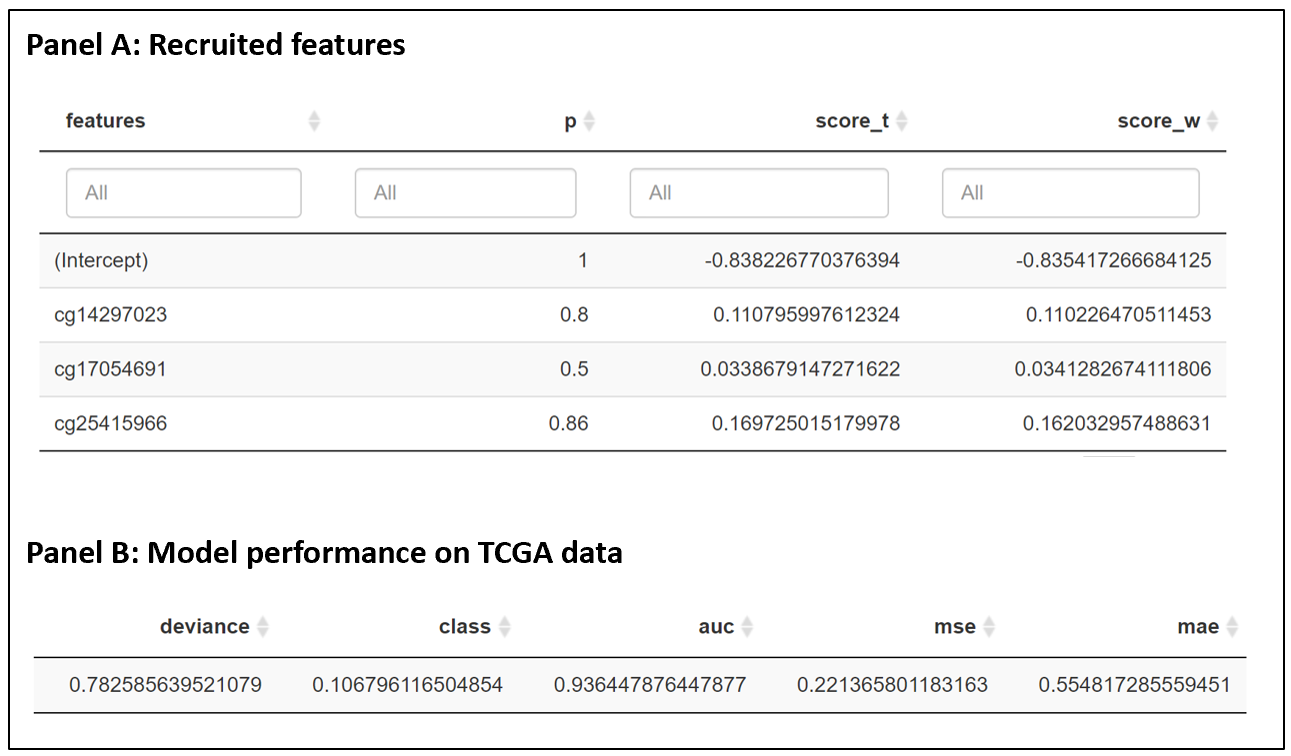


**Supplementary Table 4: other differentially methylated CpGs around the 3 CpG biomarker and their correlations with gene expression**

| CpG name | Mutated_AVG | WT_AVG | deltaBeta | CHR | UCSC_CpG_Islands_Name | MAPINFO | gene | gene expression correlation | adjusted p.value |
| --- | --- | --- | --- | --- | --- | --- | --- | --- | --- |
| cg00371593 | 0.663717003 | 0.802763028 | 0.139046 | 17 | chr17:79799286-79799624 | 79801407 | P4HB | P4HB, SIRT7 | 0.000548692, 0.008443 |
| cg19936372 | 0.769812471 | 0.874553928 | 0.1047415 | 17 | chr17:79799286-79799624 | 79801451 | P4HB | P4HB, DUS1L, PPP1R27, CCDC137 | 0.00000401, 0.00327196, 0.007596265, 0.00954 |
| cg02241160 | 0.824607834 | 0.893949592 | 0.0693418 | 17 | chr17:79799286-79799624 | 79801507 | P4HB | SIRT7 | 0.000314112 |
| cg14446575 | 0.649707789 | 0.782845826 | 0.133138 | 17 | chr17:79799286-79799624 | 79801347 | P4HB | P4HB, DUS1L | 0.00000614, 0.009386 |
| cg27570951 | 0.902120225 | 0.927136555 | 0.0250163 | 17 | chr17:79799286-79799624 | 79801578 | P4HB | - | - |
| cg15090877 | 0.803267043 | 0.854508509 | 0.0512415 | 17 | chr17:79804259-79804488 | 79801920 | P4HB | - | - |
| cg22909138 | 0.957415362 | 0.977681514 | 0.0202662 | 17 | chr17:79804259-79804488 | 79803493 | P4HB | - | - |
| cg17462329 | 0.876952402 | 0.904954465 | 0.0280021 | 17 | chr17:79804259-79804488 | 79803671 | P4HB | - | - |
| cg19219672 | 0.750772133 | 0.784446104 | 0.033674 | 17 | chr17:79804259-79804488 | 79804113 | P4HB | - | - |
| cg07955004 | 0.906510597 | 0.933377034 | 0.0268664 | 17 | chr17:79804259-79804488 | 79803515 | P4HB | - | - |
| cg19420720 | 0.863168088 | 0.929964817 | 0.0667967 | 17 | chr17:79818230-79819299 | 79816504 | P4HB | P4HB | 0.000436253 |
| cg25415966 | 0.499894708 | 0.726267302 | 0.2263726 | 20 | chr20:60961570-60962753 | 60964137 | CABLES2 | ADRM1 | 0.002592185 |
| cg07995091 | 0.681740876 | 0.778007705 | 0.0962668 | 20 | chr20:60969000-60969286 | 60966227 | CABLES2 | - | - |
| cg22451887 | 0.824745764 | 0.881353162 | 0.0566074 | 20 | chr20:60969000-60969286 | 60969143 | CABLES2 | - | - |
| cg25404458 | 0.636473989 | 0.72657488 | 0.0901009 | 20 | chr20:60969000-60969286 | 60966991 | CABLES2 | - | - |
| cg22601191 | 0.793408916 | 0.84080283 | 0.0473939 | 20 | chr20:60969000-60969286 | 60968625 | CABLES2 | - | - |
| cg14297023 | 0.663574427 | 0.85848044 | 0.194906 | 22 | chr22:36902024-36903298 | 36906998 | EIF3D | - | - |
| cg27575890 | 0.283281478 | 0.341647755 | 0.0583663 | 22 | chr22:36902024-36903298 | 36907158 | EIF3D | - | - |
| cg17054691 | 0.703830679 | 0.932620333 | 0.2287897 | 17 |  | 79813439 | P4HB | - | - |
| cg20623506 | 0.741251822 | 0.939214018 | 0.1979622 | 17 |  | 79813410 | P4HB | - | - |
| cg14492337 | 0.74375586 | 0.876057666 | 0.1323018 | 17 |  | 79813507 | P4HB | - | - |

**Supplementary Table 5: SETD2 mutation details for TCGA-KIRC:**

| Sample ID | Altered | SETD2 | SETD2: MUT | SETD2: AMP | SETD2: HOMDEL |
| --- | --- | --- | --- | --- | --- |
| TCGA-A3-3358-01 | 1 | X2413_splice (driver) | X2413_splice (driver) | no alteration | no alteration |
| TCGA-A3-3367-01 | 1 | K2545* (driver), X2477_splice (driver) | K2545* (driver), X2477_splice (driver) | no alteration | no alteration |
| TCGA-B0-4697-01 | 1 | HOMDEL (driver) | not profiled | HOMDEL (driver) | HOMDEL (driver) |
| TCGA-B0-4706-01 | 1 | X2037_splice (driver) | X2037_splice (driver) | no alteration | no alteration |
| TCGA-B0-4710-01 | 1 | W1640R | W1640R | no alteration | no alteration |
| TCGA-B0-4712-01 | 1 | Q256* (driver), K255N | Q256* (driver), K255N | no alteration | no alteration |
| TCGA-B0-4811-01 | 1 | L1778Cfs*9 (driver) | L1778Cfs*9 (driver) | no alteration | no alteration |
| TCGA-B0-4821-01 | 1 | X2037_splice (driver) | X2037_splice (driver) | no alteration | no alteration |
| TCGA-B0-4852-01 | 1 | M1627R | M1627R | no alteration | no alteration |
| TCGA-B0-5098-01 | 1 | N1541H | N1541H | no alteration | no alteration |
| TCGA-B0-5116-01 | 1 | HOMDEL (driver) | no alteration | HOMDEL (driver) | HOMDEL (driver) |
| TCGA-B0-5121-01 | 1 | Y1113* (driver) | Y1113* (driver) | no alteration | no alteration |
| TCGA-B0-5399-01 | 1 | W1782* (driver), E505* (driver), D2504* (driver) | W1782* (driver), E505* (driver), D2504* (driver) | no alteration | no alteration |
| TCGA-B0-5402-01 | 1 | S595Kfs*3 (driver) | S595Kfs*3 (driver) | no alteration | no alteration |
| TCGA-B0-5690-01 | 1 | X2475_splice (driver) | X2475_splice (driver) | no alteration | no alteration |
| TCGA-B0-5696-01 | 1 | HOMDEL (driver) | no alteration | HOMDEL (driver) | HOMDEL (driver) |
| TCGA-B0-5699-01 | 1 | S543* (driver) | S543* (driver) | no alteration | no alteration |
| TCGA-B0-5702-01 | 1 | I669* (driver) | I669* (driver) | no alteration | no alteration |
| TCGA-B2-4101-01 | 1 | E1720* (driver) | E1720* (driver) | no alteration | no alteration |
| TCGA-B2-5639-01 | 1 | D289Mfs*12 (driver) | D289Mfs*12 (driver) | no alteration | no alteration |
| TCGA-B4-5835-01 | 1 | S560* (driver), E777* (driver) | S560* (driver), E777* (driver) | no alteration | no alteration |
| TCGA-B8-A8YJ-01 | 1 | D837G | D837G | no alteration | no alteration |
| TCGA-BP-5010-01 | 1 | L1748_C1754delinsS | L1748_C1754delinsS | no alteration | no alteration |
| TCGA-BP-5169-01 | 1 | E1964* (driver) | E1964* (driver) | no alteration | no alteration |
| TCGA-BP-5178-01 | 1 | Y1666H (driver) | Y1666H (driver) | no alteration | no alteration |
| TCGA-BP-5191-01 | 1 | HOMDEL (driver) | no alteration | HOMDEL (driver) | HOMDEL (driver) |
| TCGA-BP-5198-01 | 1 | T2372Sfs*54 (driver) | T2372Sfs*54 (driver) | no alteration | no alteration |
| TCGA-CJ-4882-01 | 1 | D2004Ifs*2 (driver) | D2004Ifs*2 (driver) | no alteration | no alteration |
| TCGA-CJ-4897-01 | 1 | HOMDEL (driver) | not profiled | HOMDEL (driver) | HOMDEL (driver) |
| TCGA-CJ-4901-01 | 1 | HOMDEL (driver) | no alteration | HOMDEL (driver) | HOMDEL (driver) |
| TCGA-CJ-4920-01 | 1 | K1863Sfs*2 (driver) | K1863Sfs*2 (driver) | no alteration | no alteration |
| TCGA-CJ-4923-01 | 1 | I1194Yfs*42 (driver) | I1194Yfs*42 (driver) | no alteration | no alteration |
| TCGA-CJ-5671-01 | 1 | H1629Y | H1629Y | no alteration | no alteration |
| TCGA-CJ-5676-01 | 1 | W1782* (driver) | W1782* (driver) | no alteration | no alteration |
| TCGA-CJ-5678-01 | 1 | K466* (driver) | K466* (driver) | no alteration | no alteration |
| TCGA-CJ-5681-01 | 1 | HOMDEL (driver) | no alteration | HOMDEL (driver) | HOMDEL (driver) |
| TCGA-CJ-5682-01 | 1 | L2124* (driver) | L2124* (driver) | no alteration | no alteration |
| TCGA-CJ-5684-01 | 1 | E1528D | E1528D | no alteration | no alteration |
| TCGA-CJ-6028-01 | 1 | HOMDEL (driver) | no alteration | HOMDEL (driver) | HOMDEL (driver) |
| TCGA-CW-5580-01 | 1 | Q1368* (driver) | Q1368* (driver) | no alteration | no alteration |
| TCGA-CW-5591-01 | 1 | W1640C, HOMDEL (driver) | W1640C | HOMDEL (driver) | HOMDEL (driver) |
| TCGA-CZ-5456-01 | 1 | S618* (driver) | S618* (driver) | no alteration | no alteration |
| TCGA-CZ-5459-01 | 1 | W1827R | W1827R | no alteration | no alteration |
| TCGA-CZ-5460-01 | 1 | Y1688_L1689delins* (driver), HOMDEL (driver) | Y1688_L1689delins* (driver) | HOMDEL (driver) | HOMDEL (driver) |
| TCGA-CZ-5461-01 | 1 | R1625C (driver) | R1625C (driver) | no alteration | no alteration |
| TCGA-CZ-5464-01 | 1 | HOMDEL (driver) | not profiled | HOMDEL (driver) | HOMDEL (driver) |
| TCGA-CZ-5470-01 | 1 | R973* (driver) | R973* (driver) | no alteration | no alteration |
| TCGA-DV-A4VX-01 | 1 | Y2296Lfs*72 (driver) | Y2296Lfs*72 (driver) | no alteration | no alteration |
| TCGA-EU-5904-01 | 1 | HOMDEL (driver) | no alteration | HOMDEL (driver) | HOMDEL (driver) |
| TCGA-G6-A8L6-01 | 1 | P2380Tfs*31 (driver) | P2380Tfs*31 (driver) | no alteration | no alteration |

**SUPPLEMENTARY FIGURES**

**Supplementary figure 1:** Genomic distribution of hypomethylated and hypermethylated CpGs in SETD2 alternative variant cases, in (A) uterine cancer, (B) mesothelioma, (C) clear cell renal cell carcinoma (D) skin cutaneous melanoma (E) lung adenocarcinoma (F) bladder cancer (G) kidney renal papillary cancer (H) cervical squamous cell carcinoma (I) stomach adenocarcinoma (J) liver hepatocellular carcinoma (K) lung squamous cell carcinoma (L) prostate adenocarcinoma (M) colorectal cancer

**
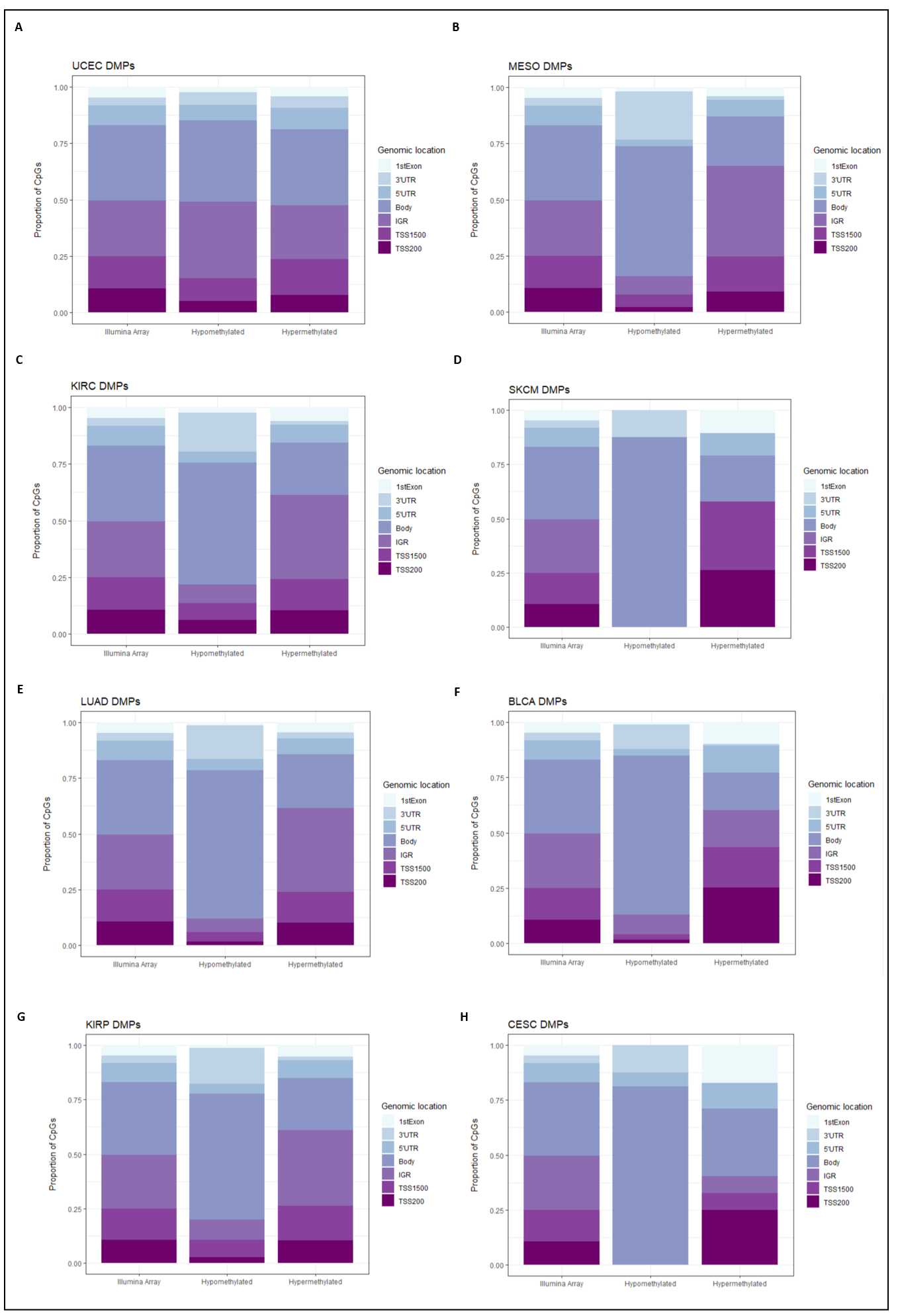
**

(continued Supplementary figure 1)

**
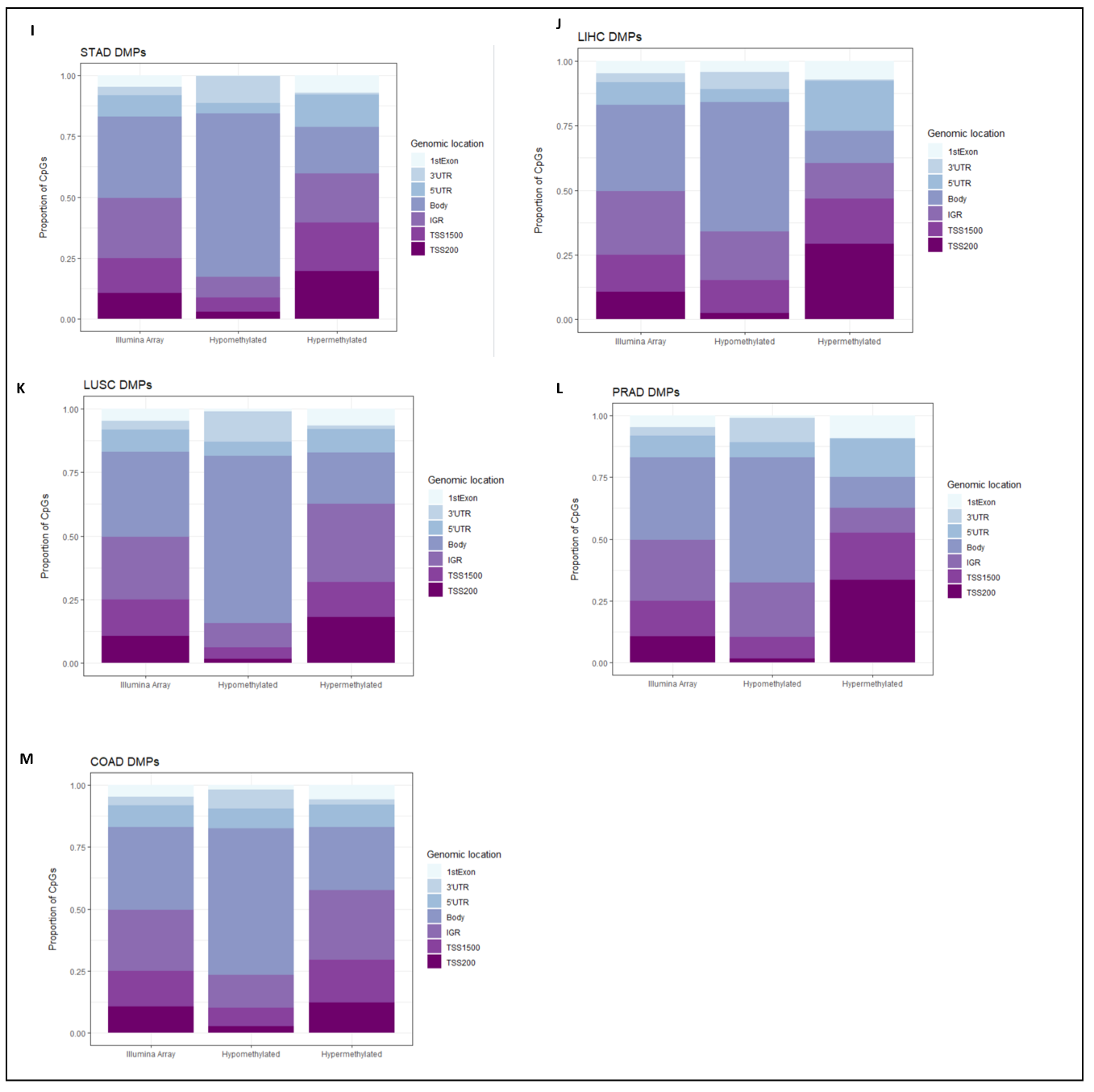
**

**Supplementary figure 2:** Genomic distribution of hypomethylated and hypermethylated CpGs in SETD2 low expressing cases from (A) diffuse large B-cell lymphoma, (B) esophageal cancer, (C) head and neck squamous cell carcinoma, (D) kidney chromophobe, (E) thyroid carcinoma, (F) sarcoma, (G) glioblastoma multiforme, (H) pheochromocytoma and paraganglioma


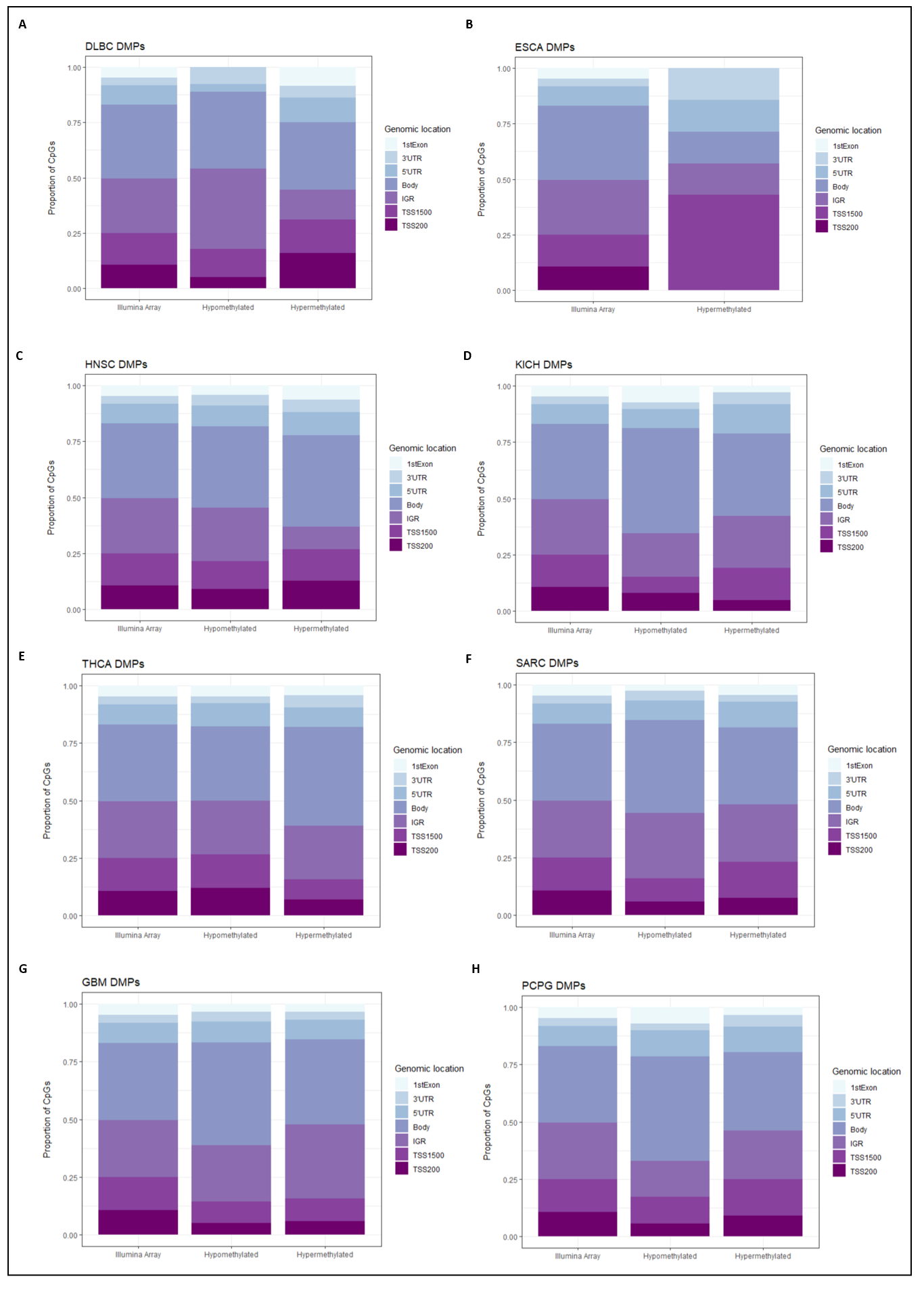


**Supplementary figure 3: representative figures of the distribution of H3K36me3 around the most hypomethylated CpGs in renal cancer in SETD2 WT renal cancer cell line and SETD2 knock-outs (data from Tiedemann et al 2016)**

**(A)**


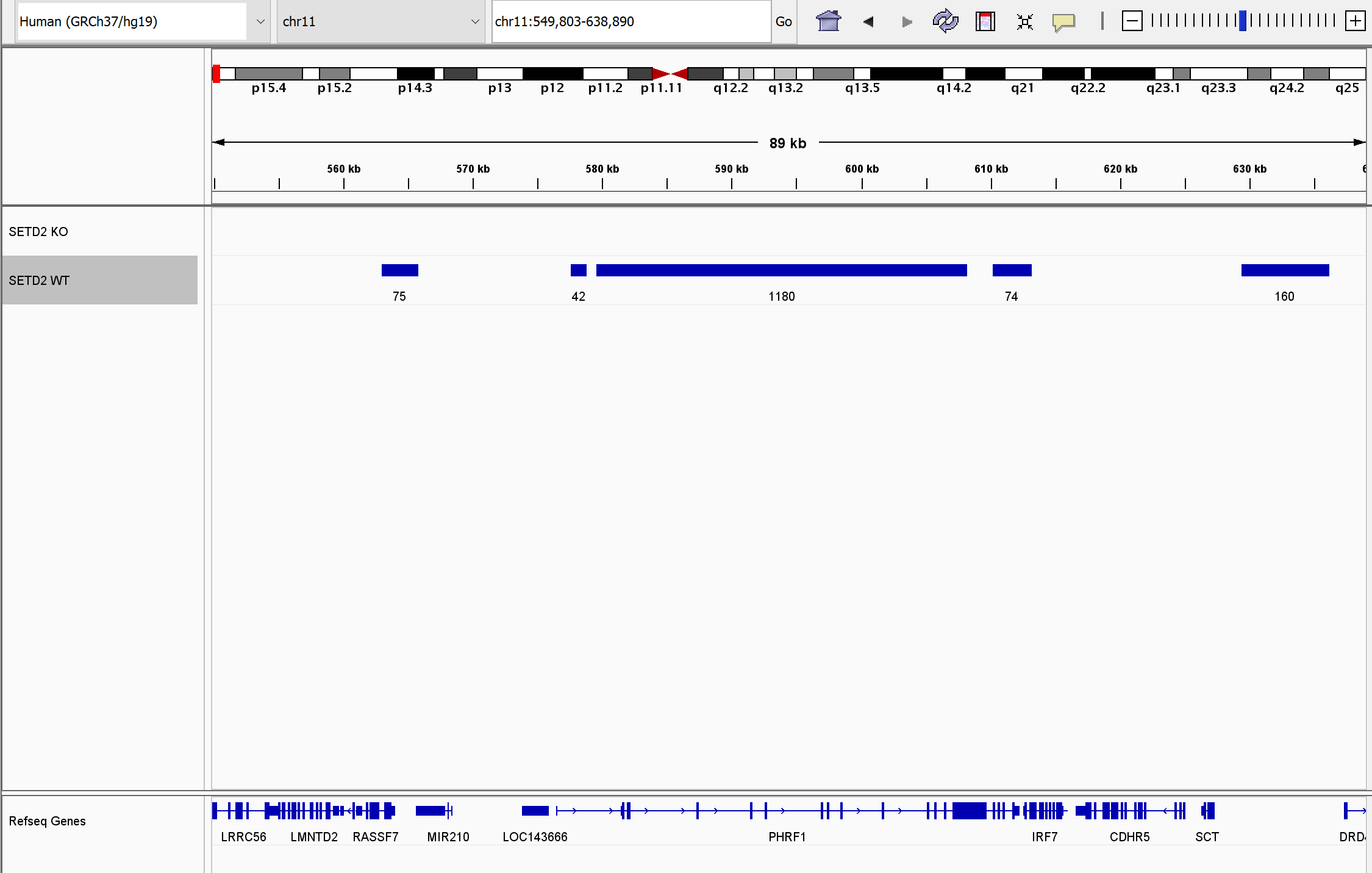


**(B)**


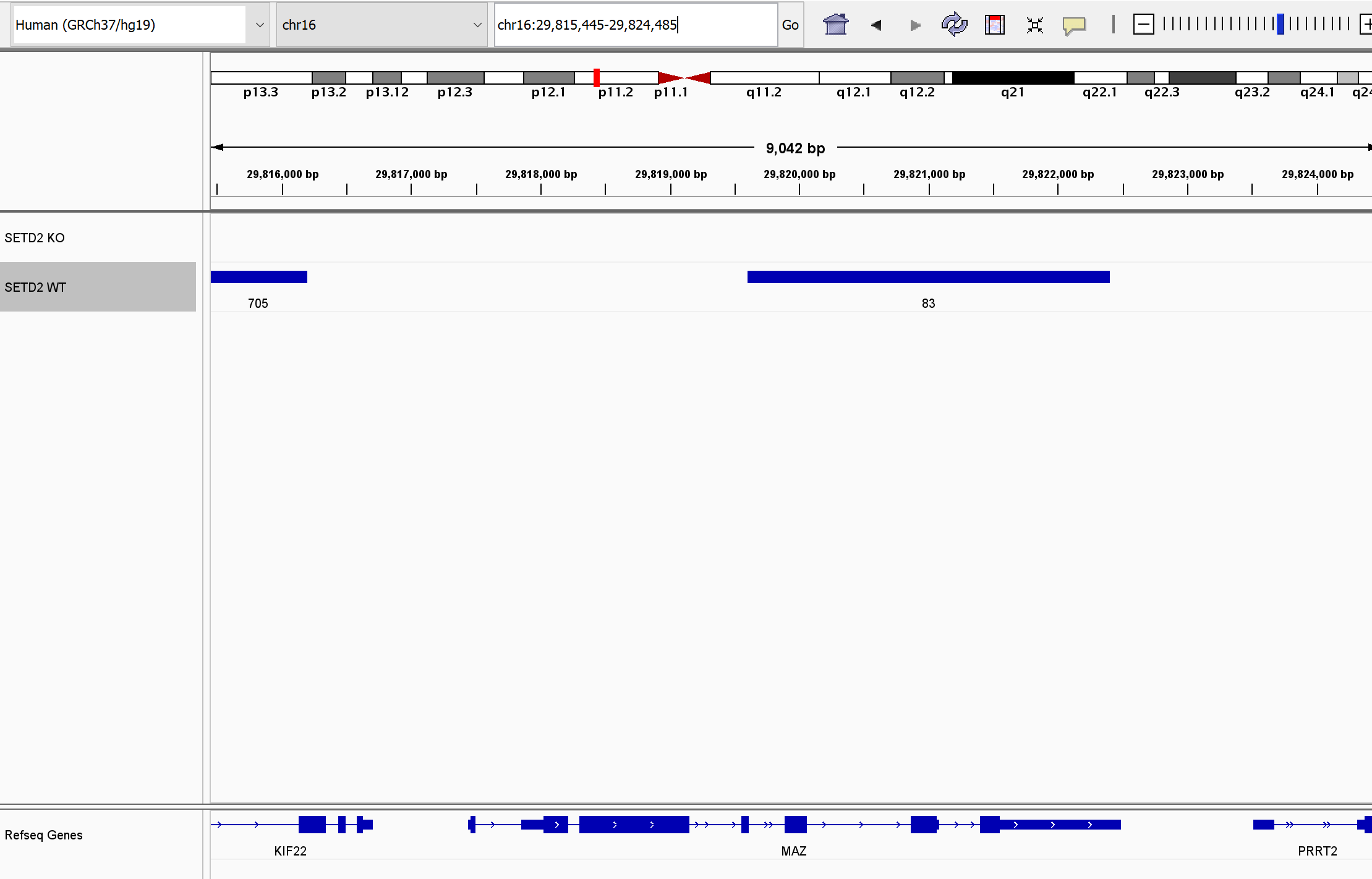


**(C)**


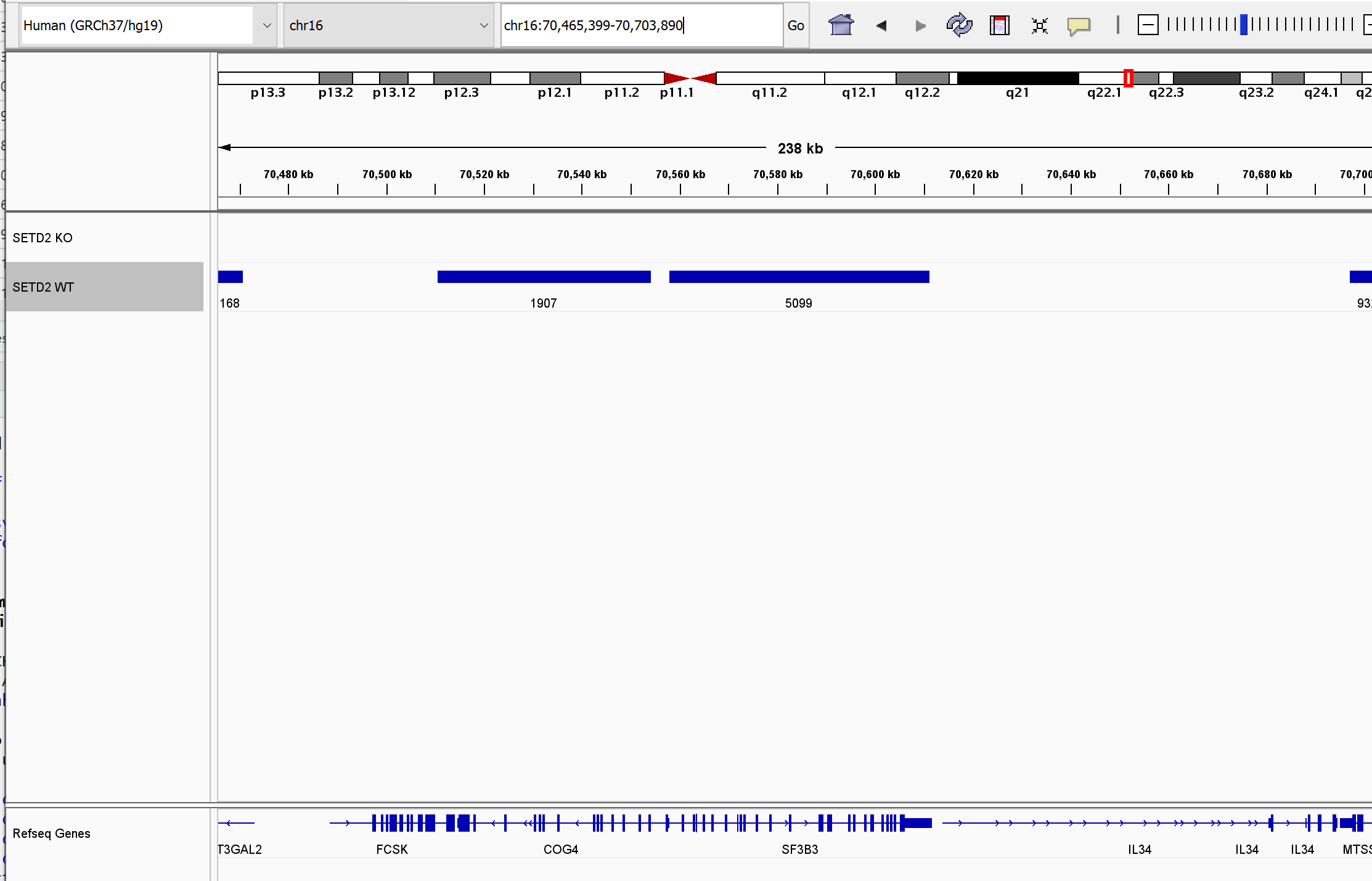


**(D)**


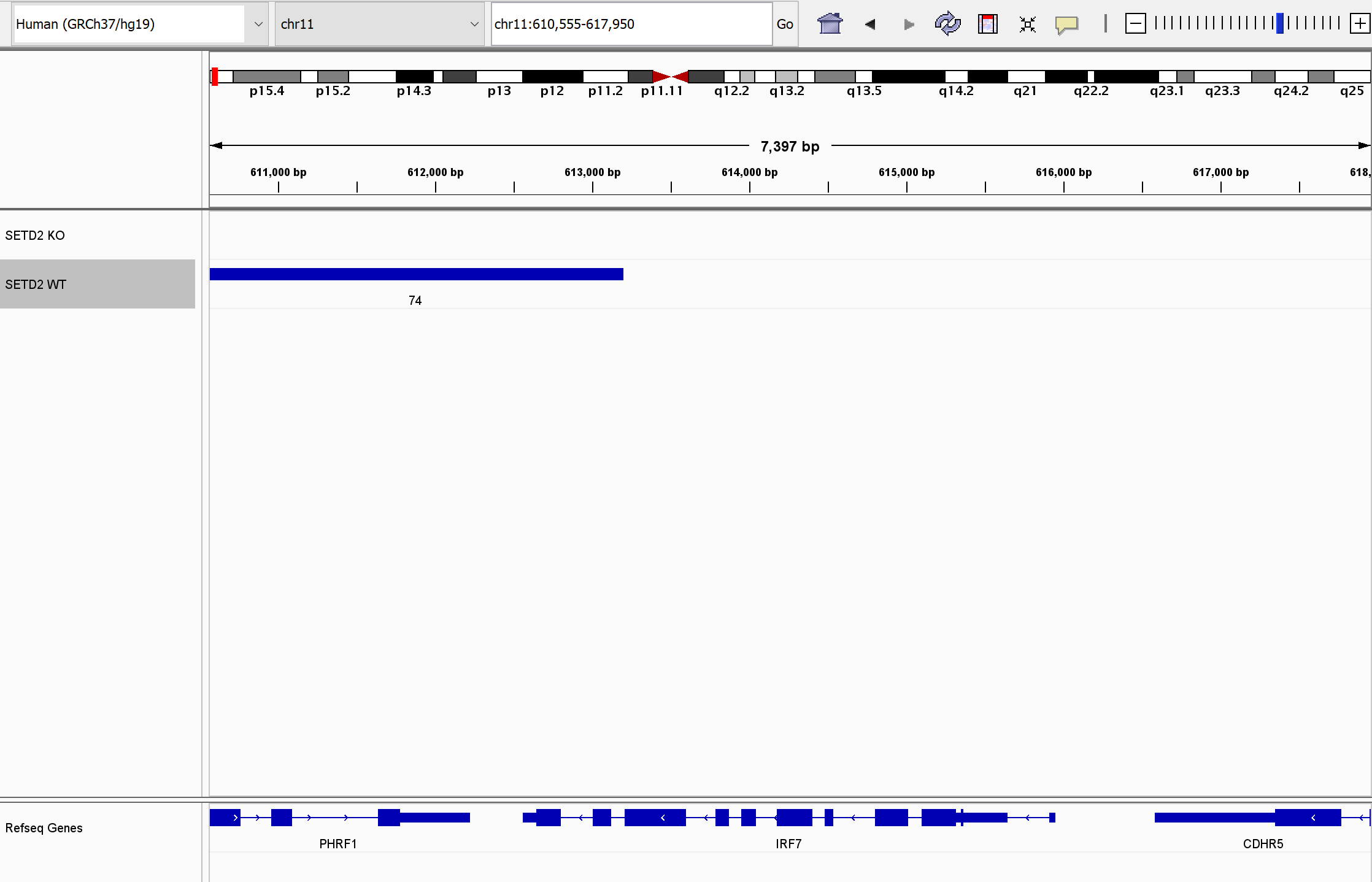


**Supplementary figure 4: representative figures of the distribution of H3K36me3 around the most hypermethylated CpGs in renal cancer in SETD2 WT renal cancer cell line and SETD2 knock-outs (data from Tiedemann et al 2016)**

**(A)**


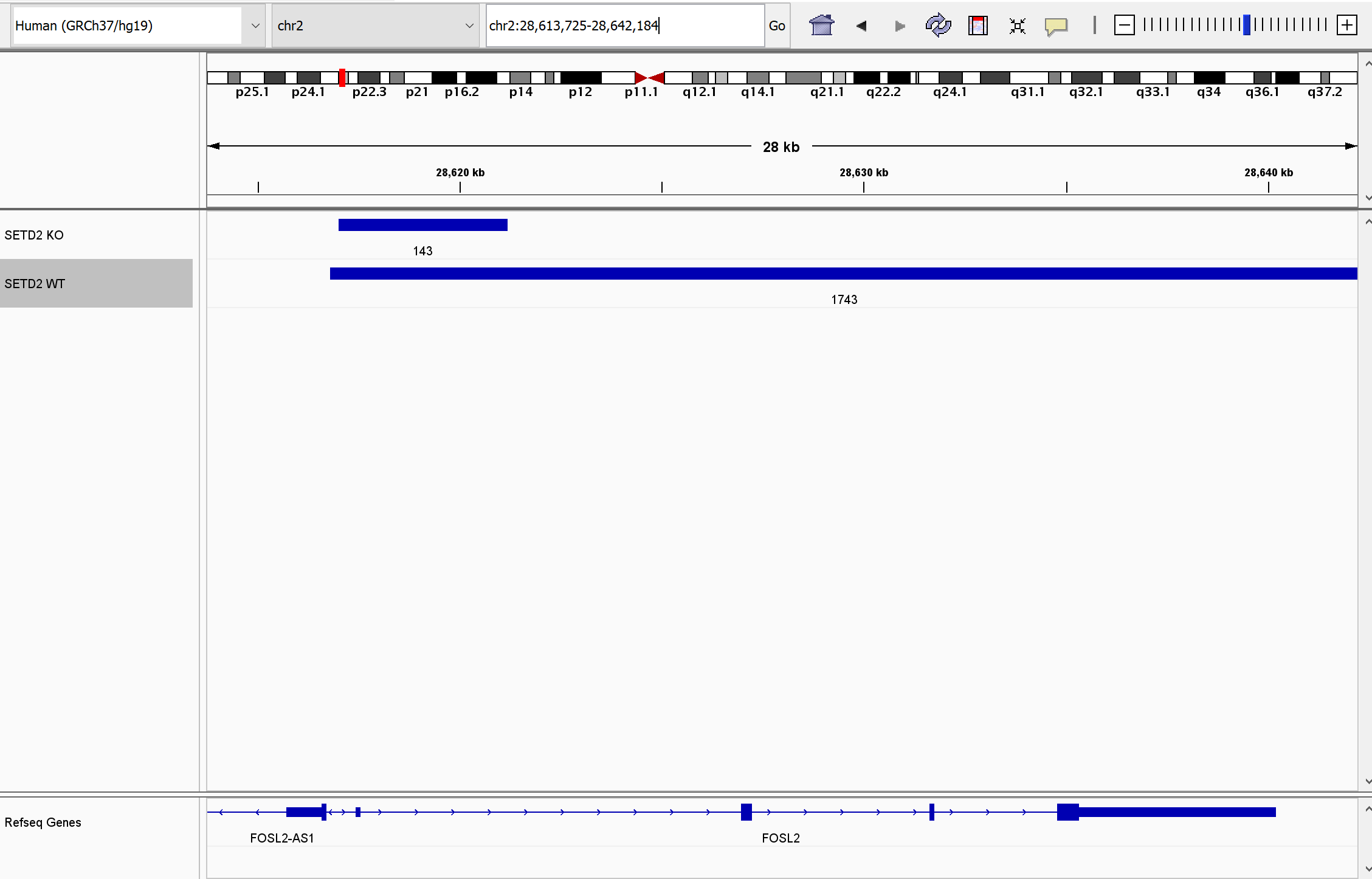


**(B)**


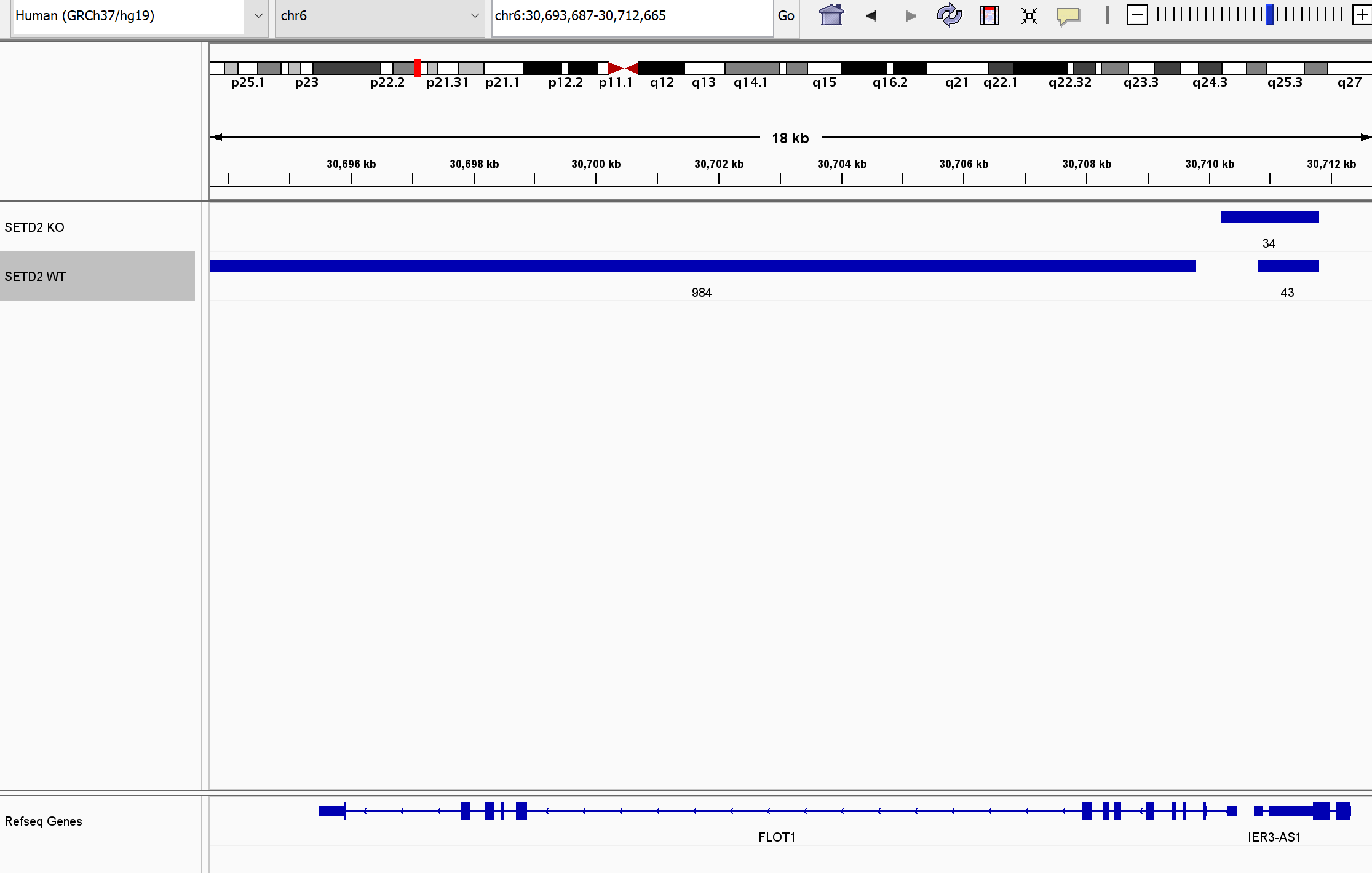


**(C)**


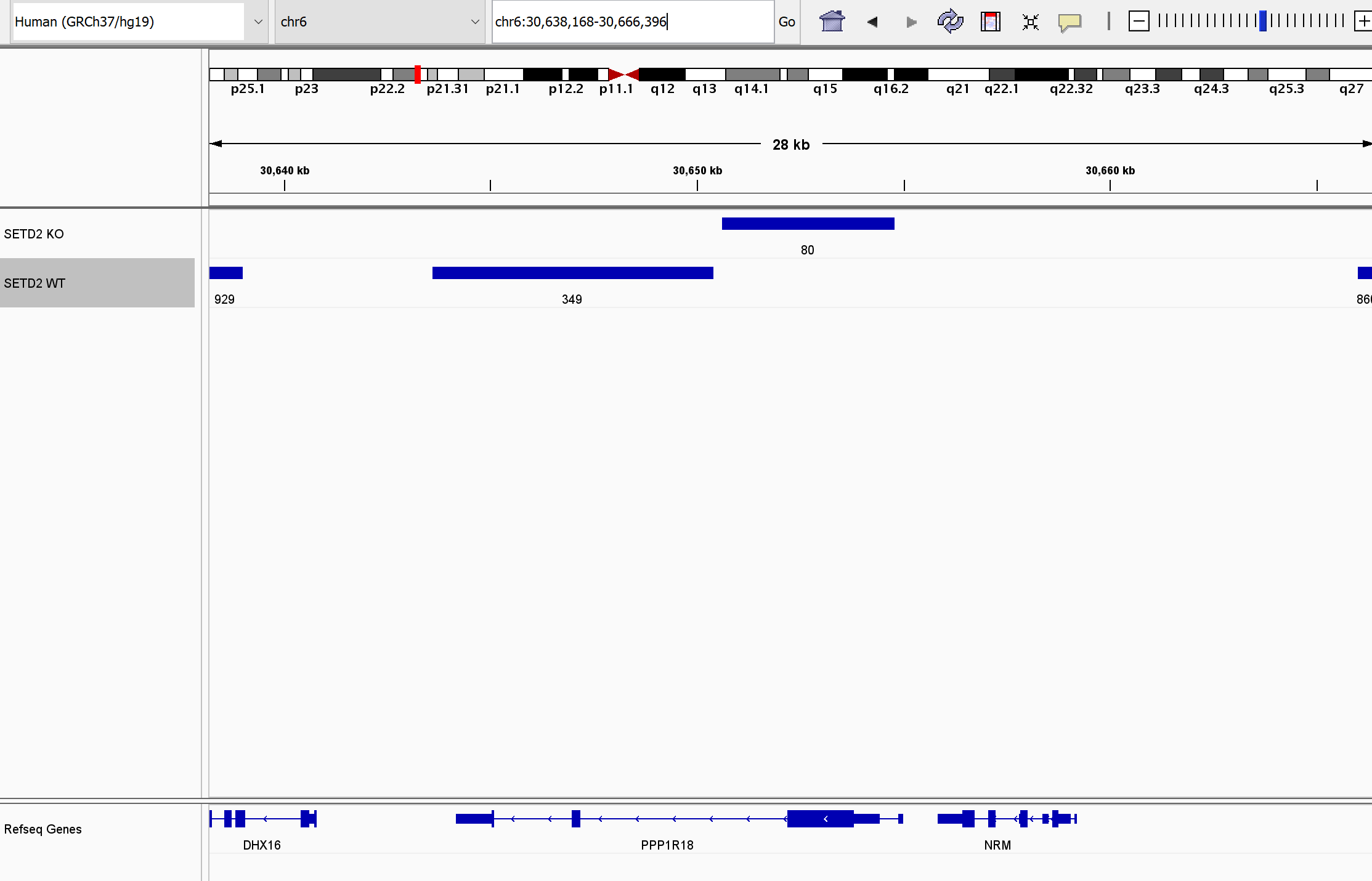


**(D)**


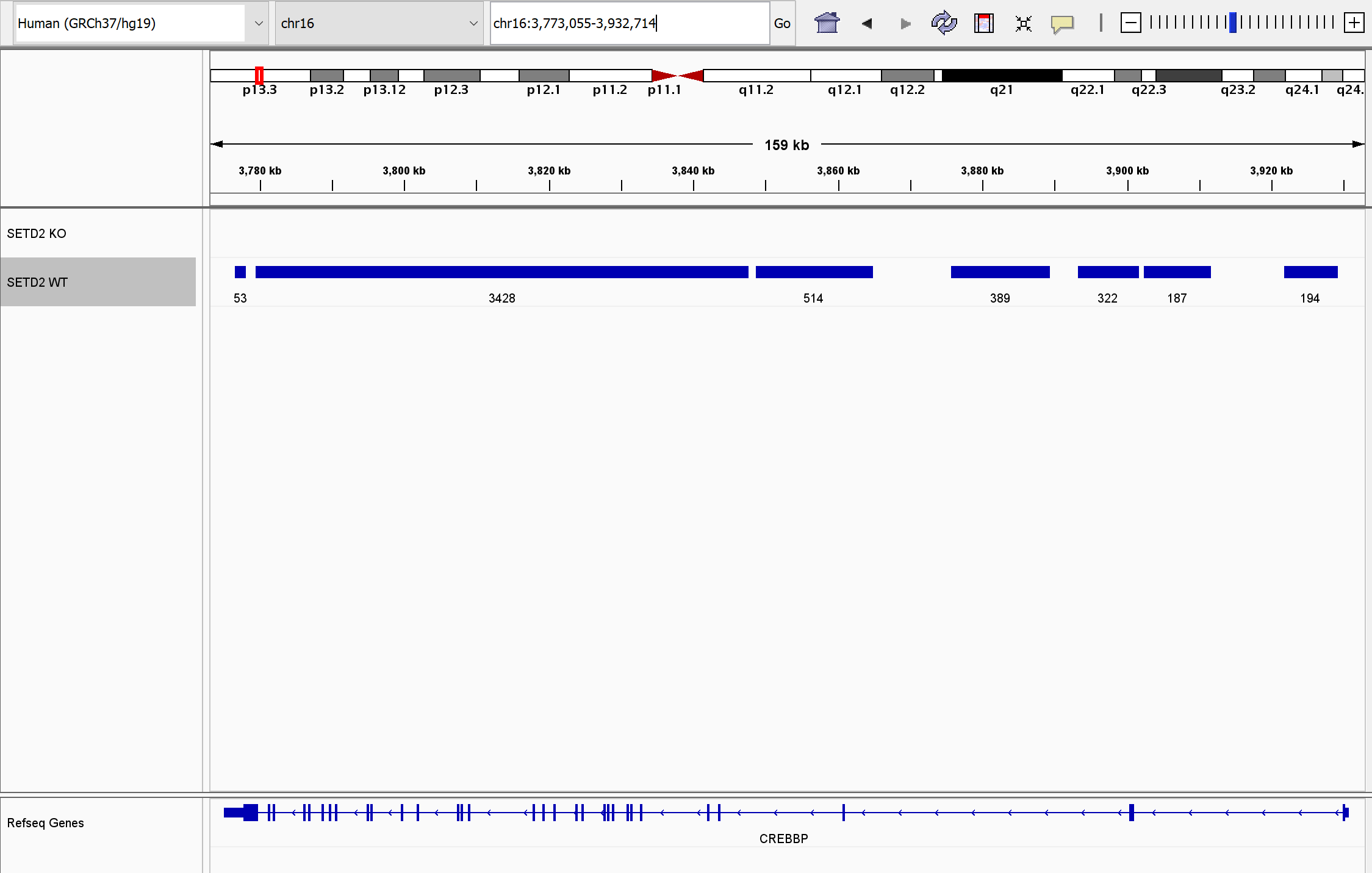


**Supplementary figure 5:**

No significant difference exists between the tumour stage in SETD2 WT (unaltered) and SETD2 mutated (altered) groups.


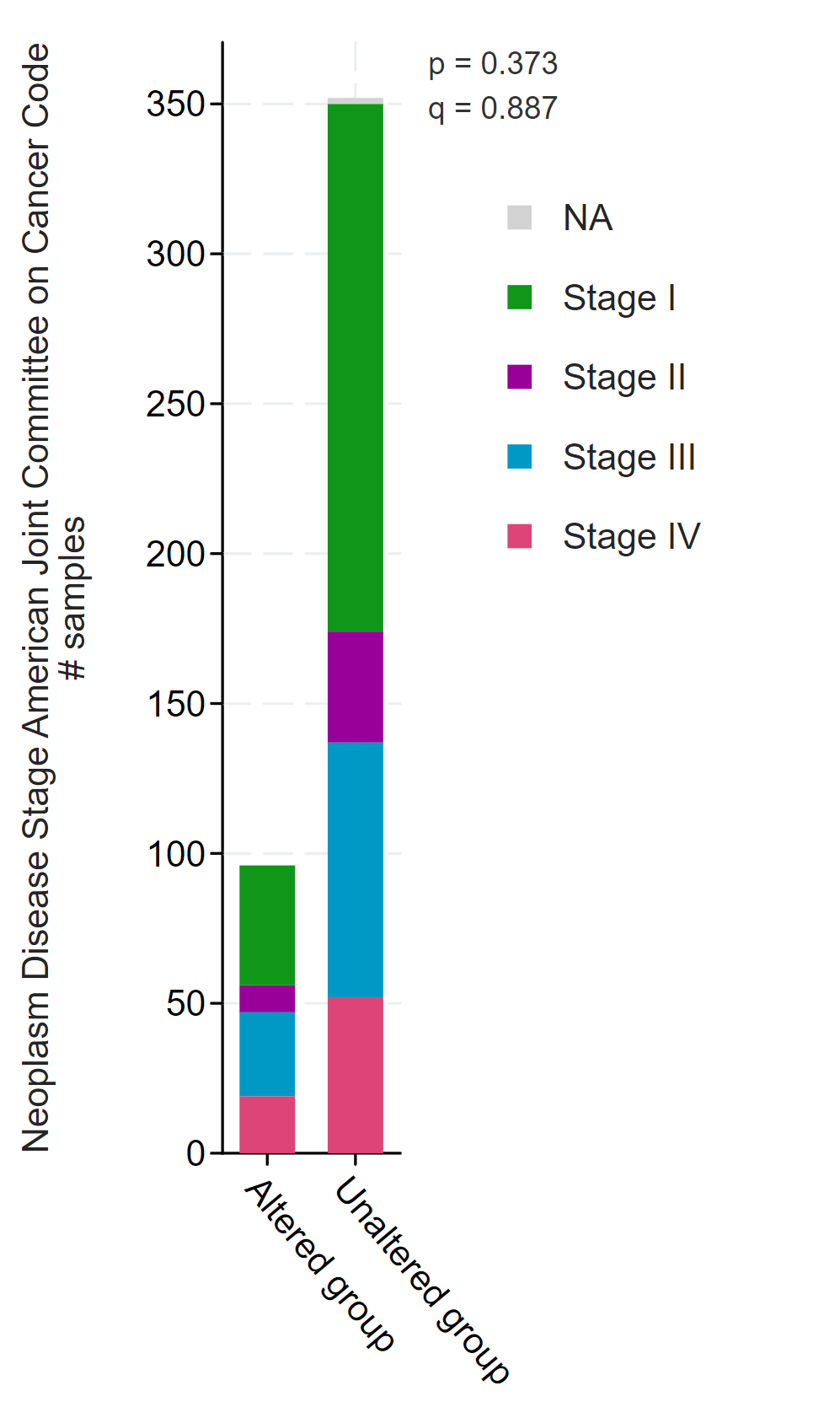

Supplement: Supplementary file 1 — Supplementary Material 1 [file 12885_2023_11162_MOESM1_ESM.docx]
